# DNA demethylation affects imprinted gene expression in maize endosperm

**DOI:** 10.1186/s13059-022-02641-x

**Published:** 2022-03-09

**Authors:** Qiang Xu, Leiming Wu, Zhixiang Luo, Mei Zhang, Jinsheng Lai, Lin Li, Nathan M. Springer, Qing Li

**Affiliations:** 1 National Key Laboratory of Crop Genetic Improvement, Huazhong Agricultural University, Wuhan, 430070 China; 2 Key Laboratory of Plant Molecular Physiology, Institute of Botany, Chinese Academy of Sciences, Nanxincun 20, Fragrant Hill, Beijing, 100093 China; 3 State Key Laboratory of Plant Physiology and Biochemistry and National Maize Improvement Center, Department of Plant Genetics and Breeding, China Agricultural University, Beijing, 100094 China; 4 Department of Plant and Microbial Biology, University of Minnesota, St. Paul, MN 55108 USA; 5 Hubei Hongshan Laboratory, Wuhan, 430070 China

**Keywords:** DNA demethylation, Imprinting, Tissue-specific expression, Transcription factor binding, Maize

## Abstract

**Background:**

DNA demethylation occurs in many species and is involved in diverse biological processes. However, the occurrence and role of DNA demethylation in maize remain unknown.

**Results:**

We analyze loss-of-function mutants of two major genes encoding DNA demethylases. No significant change in DNA methylation has been detected in these mutants. However, we detect increased DNA methylation levels in the mutants around genes and some transposons. The increase in DNA methylation is accompanied by alteration in gene expression, with a tendency to show downregulation, especially for the genes that are preferentially expressed in endosperm. Imprinted expression of both maternally and paternally expressed genes changes in F_1_ hybrid with the mutant as female and the wild-type as male parental line, but not in the reciprocal hybrid. This alteration in gene expression is accompanied by allele-specific DNA methylation differences, suggesting that removal of DNA methylation of the maternal allele is required for the proper expression of these imprinted genes. Finally, we demonstrate that hypermethylation in the double mutant is associated with reduced binding of transcription factor to its target, and altered gene expression.

**Conclusions:**

Our results suggest that active removal of DNA methylation is important for transcription factor binding and proper gene expression in maize endosperm.

**Supplementary Information:**

The online version contains supplementary material available at 10.1186/s13059-022-02641-x.

## Background

Abundant heritable phenotypic diversity occurs between and within species. This diversity is largely due to variation in heritable information and its complex interaction with the environment. It is clear that DNA contains much of the heritable information. However, other types of heritable epigenetic information, including DNA methylation, are beginning to be recognized in the past few decades [1]. DNA methylation is a phenomenon where the methyl group is added onto the 5′ position of the cytosine. It occurs in both animals and plants [2]. One main property of DNA methylation is that it is reversible and dynamic, providing a way for organisms to respond to both endogenous and exogenous signals.

Several pathways are involved in the dynamic regulation of DNA methylation levels [1]. DNA methylation is de novo established by RNA-directed DNA methylation pathway (RdDM). Once it is established, it can be maintained by different pathways depending on which sequence context DNA methylation occurs. The CG and CHG (H = A, C or T) methylation is maintained by methyltransferase 1 (MET1) and chromomethylase 3 (CMT3), respectively. The CHH methylation can be maintained by either the RdDM or CMT2 pathway [3, 4]. Removal of DNA methylation usually occurs in two pathways, the passive and active pathway. DNA methylation can be passively lost after several rounds of cell division if it cannot be efficiently maintained. DNA methylation can also be actively removed by catalytic enzymes [2].

A subset of plant glycosylase domain containing proteins can act as enzymes to remove methylated cytosines in plants. There are four genes in the *Arabidopsis* genome encoding proteins with DNA demethylase activities, including *DEMETER* (*DME*), *REPRESSOR OF SILENCING 1* (*ROS1*, also known as *DML1*), *DML2*, and *DML3* [5, 6]. Among the four genes, *DME* and *ROS1* are the main players. The *ROS1* gene is mainly expressed in vegetative tissues while the *DME* gene is mainly expressed in reproductive tissues, especially in the central cell of the female gametophyte and the vegetative cell of the male gametophyte [7–9]. In addition to these demethylase, there are many other proteins involved in DNA demethylation, including proteins that guide the demethylase to the target regions [10, 11]. Besides *Arabidopsis*, DNA demethylation has been reported in many other species, including tomato, wheat, rice, Medicago, and strawberries [12–16], but it has not been characterized in maize.

Previous studies found that only a small portion of the genome is sensitive to mutations of the genes involved in DNA demethylation. For example, only 6902 regions were found to be targeted by ROS1 in *Arabidopsis* [17]. Therefore, an interesting question is which regions are specifically targeted. Tang et al. analyzed ROS1 targets in *Arabidopsis* and found that they are enriched for histone markers H3K27me3 and H3K18Ac and depleted for H3K9me2. These ROS1-targeted regions are usually enriched for transposable elements (TEs) and intergenic regions [17]. Similarly, a study in tomato identified ~30,000 regions that are targeted by ROS1 and these regions tend to be TEs and intergenic regions [12]. The larger number of ROS1 targets in tomato compared to *Arabidopsis* is probably due to the differences in the size and TE compositions between the two genomes. Maize, where transposons were first identified, is estimated to have 85% of its 2500-Mb genome to be TE [18]. Considering the preference of ROS1 for TEs and the high TE content in maize, it is proposed that the ROS1 targets in maize are more abundant and ROS1 in maize may have important roles in regulation of gene expression and phenotypes [12].

One major role of DNA demethylation is to control imprinted gene expression. Imprinting is a phenomenon where the allele-specific expression of genes is dependent on parent-of-origin. This includes many examples of maternally expressed genes (MEGs) that are often only expressed in endosperm tissue as well as paternally expressed genes (PEGs) that are often expressed in multiple tissues [19–22]. It was first discovered in maize [23] and occurs in both animals and plants. Some imprinted genes are conserved among different species [24]. Imprinting predominantly occurs in endosperm probably due to the fact that endosperm does not contribute to the next generation and thus there is no need for the expressed allele to be silenced or vice versa in the next generation [25]. At least some imprinted maize genes are associated with allelic changes in DNA methylation [26] and mutants in the DME demethylase in *Arabidopsis* have substantially altered imprinting [5, 25, 27]. In maize, hundreds of imprinted genes have been identified [19–22]. However, the mechanisms controlling the occurrence of imprinting in maize are unknown.

Here we investigated the function of two genes (*ZmROS1a* and *ZmROS1b*, abbreviated as *ZmROS1ab*) encoding DNA demethylases in maize. We found that ZmROS1ab preferentially target regions with regulatory function in gene expression. ZmROS1ab is important for the proper expression of genes in endosperm, including imprinted genes. Finally, we showed that the regulation of ZmROS1ab on gene expression is partly dependent on DNA methylation-sensitive binding of transcription factor. Our results suggest a critical role and a possible mechanism of ZmROS1ab in regulating gene expression in maize kernel.

## Results

### Characterization of the single and double mutants of *ZmROS1a* and *ZmROS1b*

To study the role of DNA demethylation in maize, we first identified genes encoding DNA demethylase in maize. To this end, the four DNA demethylase genes in *Arabidopsis*, namely *DME*, *ROS1*, *DML2*, and *DML3* were used as queries against the maize B73 reference genome (version 4). Four genes with high similarity were identified and were named *ZmROS1a - 1d*. The four maize genes encode proteins with similar domains, including the catalytic DNA glycosylase domain and a RRM-fold domain at the C-terminal with uncharacterized function (Additional file 1: Fig. S1). Phylogenetic analysis showed relatively weak support to classify the maize genes relative to specific *Arabidopsis* demethylase genes (Additional file 1: Fig. S1b). ZmROS1b and ZmROS1d are closely related to each other and have two homologs in rice. ZmROS1a is closely related to three other rice demethylase genes while ZmROS1c does not have closely related sequences in rice (Additional file 1: Fig. S1b). The four genes show different expression levels and patterns across tissues and developmental stages (Additional file 1: Fig. S1c). In general, they have higher expression in reproductive tissues and lower expression in vegetative tissues. The *ZmROS1a* and *ZmROS1b* genes are the two most highly expressed copies, especially in embryo and endosperm, with *ZmROS1a* being the predominantly expressed copy in embryo and *ZmROS1b* in endosperm. Therefore, the subsequent analyses focus on these two genes.

**Fig. 1:**
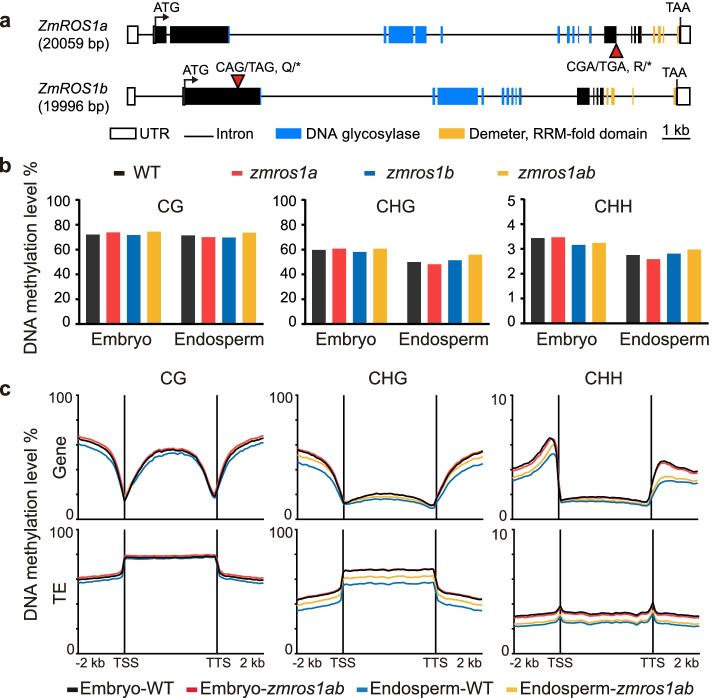
Comparison of DNA methylation levels between WT and mutants. **a** Gene structure of *ZmROS1a* and *ZmROS1b*. **b** Global DNA methylation levels in mutants and WT. **c** DNA methylation levels around genes (upper panel) and TEs (down panel) for CG, CHG, and CHH

We obtained nonsense EMS mutants of *ZmROS1a* and *ZmROS1b* in the B73 background (Fig. 1). Both mutants have a single-nucleotide mutation that introduces a premature stop codon. The truncated proteins lack the RRM-fold domain in ZmROS1a and both the DNA glycosylase and RRM-fold domains in ZmROS1b. In *zmros1a* single mutant plants, expression of the *ZmROS1a* gene was significantly reduced, while no difference was observed for the other three genes (Additional file 1: Fig. S2). Similarly, expression of *ZmROS1b* was significantly reduced in *zmros1b* single mutant, and the other three genes show no (*ZmROS1c* and *ZmROS1d*) or slightly reduced (*ZmROS1a*) expression. The double mutant (*zmros1ab*) was obtained by crossing these two single mutants and identifying plants that are homozygous mutant for both genes. In the homozygous mutant, expression of both the *ZmROS1a* and *ZmROS1b* gene was significantly reduced (Additional file 1: Fig. S2a). Phenotypic analysis revealed that the double mutant had reduced height and flowered later than the wild-type (Additional file 1: Fig. S2b-e). The double mutant is viable and produces ears with normal to slightly reduced fertility (Additional file 1: Fig. S2f, S2g). Overall, the phenotype of the single mutants is relatively subtle compared to double mutant, suggesting functional redundancy of these two genes.

We further characterized the transmission of the two mutations in single mutants or in the double mutant (Additional file 1: Fig. S2h-j). For both single mutants, the homozygous mutant kernels in the self-pollinated ear of the heterozygous plant appear at an expected ratio of 1/4. Similarly, the double homozygous mutants appear at the expected ratio of 1/16 in the self-pollinated ear of the *zmros1a*/+;*zmros1b*/+ plant. However, *zmros1ab* double mutant has reduced seed set and kernel weight (Additional file 1: Fig. S2f, S2g), suggesting reduced vigor of the double mutant plants.

### Global DNA methylation patterns in mutants of *ZmROS1ab*

We performed whole-genome bisulfite sequencing using embryo and endosperm 14 days after pollination in wild-type (WT) and the mutant genotypes (Additional file 2). These tissue stages were chosen because both *ZmROS1a* and *ZmROS1b* have high expression levels in these tissues at this stage (Additional file 1: Fig. S1c). Globally, the DNA methylation levels in all three sequence contexts (CG/CHG/CHH) are quite similar between WT and the single and double mutants in both embryo and endosperm (Fig. 1b, Additional file 1: Fig. S3, Additional file 2), suggesting that loss of *ZmROS1ab* does not lead to major changes in genome-wide DNA methylation levels.

We proceeded to assess DNA methylation levels around genes and transposons. The metaprofiles around genes and transposons show slightly lower levels of DNA methylation in endosperm compared to embryo tissue in WT (Fig. 1c) In embryo tissue, there is very little difference in context-specific DNA methylation levels in the mutants relative to WT (Fig. 1c, Additional file 1: Fig. S4). However, in endosperm tissue the mutant has higher levels of DNA methylation compared to WT, in both CG and CHG contexts (Fig. 1c, Additional file 1: Fig. S4). Interestingly, the analysis of specific TE superfamilies reveals that the change in methylation levels in the double mutant is more pronounced for a subset of TE families (Additional file 1: Fig. S5, labeled as red star). Together, these results suggest that maize ZmROS1ab does not target the genome equally and may play a role in the reduced methylation levels observed in endosperm relative to other tissues in a locus-dependent manner.

### ZmROS1ab-dependent demethylation in endosperm at thousands of loci

**Fig. 2:**
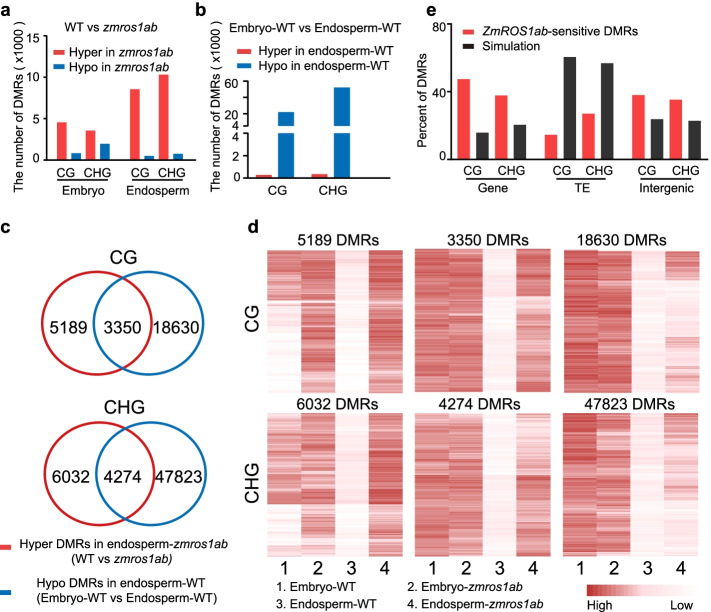
Characterization of *zmros1ab* DMRs. **a** DMRs between WT and *zmros1ab* mutant in embryo and endosperm. **b** DMRs between embryo and endosperm in WT. **c** Overlap of DMRs in **a** with DMRs in **b**. **d** Heatmap to show DNA methylation levels of the shared or unique DMRs from **c**. **e** Genomic feature of “*ZmROS1ab*-sensitive” DMRs. The simulation represents 10,000 control windows that were randomly selected from the 100-bp windows meeting the following criteria: having at least 6 cytosine sites and at least 3X coverage in both WT and mutant

To determine the genomic targets of ZmROS1ab, we first identified differentially methylated regions (DMRs) between mutants and WT in embryo and endosperm. In total, 1108–11,090 DMRs were identified in each mutant for each of the three sequence contexts (Additional file 1: Table S1, Additional file 3). Consistent with the genome-wide DNA methylation levels, more DMRs were identified in the endosperm than in embryo, and more hypermethylated DMRs (mutant > WT) were identified in the mutants (Fig. 2, Additional file 1: Table S1). Some DMRs were shared among the two single mutants and the double mutant while others were only identified in a subset of the mutant genotypes (Additional file 1: Fig. S6). The DMRs that were only identified in the double mutant generally show differences in the expected direction in the single mutants though not reaching the criteria for being classified as a DMR. For example, 6050 CG DMRs were only identified in *zmros1ab* endosperm; however, these DMRs also show increased methylation levels in both single mutants compared to WT (Additional file 1: Fig. S6b). Interestingly, similar methylation levels for these non-shared DMRs were observed between the two single mutants in embryo, while significant higher methylation levels were observed for *zmros1b* mutant in endosperm (Additional file 1: Fig. S6b). This is consistent with the highest expression of *ZmROS1b* in endosperm among the four copies and suggests a functional divergence of the *ZmROS1a* and *ZmROS1b* genes across different tissues. In summary, thousands of loci were sensitive to *zmros1ab* mutation with a tendency to show increased DNA methylation.

To further determine the potential role of ZmROS1ab in the observed hypomethylation in endosperm tissue relative to embryo (Fig. 1c), we identified DMRs between embryo and endosperm in WT and asked how these DMRs overlap with the DMRs in endosperm between *zmros1ab* mutant and WT. In total, 22,264 and 52,450 DMRs were identified between embryo and endosperm for CG and CHG respectively. The majority (99% for both CG and CHG DMRs) of these DMRs show hypomethylation in the endosperm relative to the embryo (Fig. 2b). There are a small number of DMRs that show higher methylation levels in endosperm than in embryo, similar to observations in prior studies [28, 29]. A comparison between the hypomethylated DMRs (endosperm < embryo) and the hypermethylated endosperm DMRs (*zmros1ab* > WT) revealed that 39% of CG (3350/8539) and 41% of CHG (4274/10306) hypermethylated DMRs identified in mutant compared to WT endosperm are also endosperm versus embryo DMRs (Fig. 2c). This shared subset of DMRs (3350 CG and 4274 CHG) showed reduced methylation in WT endosperm (compared to WT embryo) but not in *zmros1ab* endosperm (Fig. 2d), suggesting a role of ZmROS1ab in mediating endosperm hypomethylation. The non-shared sets include some DMRs with similar methylation patterns as shared DMRs (Fig. 2d). For example, among the 5189 CG DMRs that are only identified in the WT versus *zmros1ab* comparison, about 1/3 of them show clear hypomethylation in WT endosperm compared to embryo though not reaching the stringent criteria to be defined as DMRs (Fig. 2d). The non-shared set also include DMRs that are hypomethylated in both WT and *zmros1ab* endosperm compared to WT embryo (Fig. 2d), suggesting a possible role of the other two DNA glycosylases in endosperm hypomethylation. The subsequent analyses will focus on the shared set (3350 CG DMRs and 4274 CHG DMRs) which represents high-confident regions where ZmROS1ab is required to remove the DNA methylation in endosperm (hereafter referred to “*ZmROS1ab*-sensitive DMRs”).

The *ZmROS1ab*-sensitive DMRs are enriched around genes and depleted in transposons (Fig. 2e). However, there are still 15% CG and 27% CHG DMRs that were located in transposons probably due to the fact that transposons comprise a large proportion of the genome (Fig. 2e). When examining the distribution of DMRs around genes, an enriched peak was observed in the region immediately upstream of the transcriptional start site (Additional file 1: Fig. S7). For the DMRs that are located far away from genes, including these located within transposons or intergenic regions, they are located closer to genes than random controls (Additional file 1: Fig. S7b). By exploiting the 3D genome and histone modification data [30], we found that 255 (14.5%) CG and 140 (5.3%) CHG DMRs are overlapping regions that form chromatin loops with genes, and these percentages are higher than random controls (Additional file 1: Fig. S7c). To look at whether these DMRs with chromatin loops are enriched for binding sites of any transcription factors (TFs), we used public data of 104 TFs [31]. The pattern is quite similar for all 104 TFs. One example is shown in Additional file 1: Fig. S7d. There is no enrichment of TF binding sites for the CG and CHG DMRs. For CHH DMRs, the binding seems to be higher on the flanking regions of the DMR. Since this pattern was observed for all 104 TFs, it is quite likely that this is not biologically meaningful. Together, these results suggest that many ZmROS1ab targets (the “*ZmROS1ab*-sensitive DMRs”) occur within regions that may be important for gene regulation.

### ZmROS1ab regulates endosperm gene expression

To explore the role of ZmROS1ab in regulating gene expression, we performed RNA-seq on WT and *zmros1ab* mutant using the same tissues that were used for DNA methylation analysis (Additional file 2). Principal component analysis showed that the samples can be separated into four groups that are separated by genotype (WT vs mutant) and tissues (embryo vs endosperm). The three biological replicates clustered closely, suggesting high quality of the RNA-seq data (Additional file 1: Fig. S8). The expression of both *ZmROS1a* and *ZmROS1b* were reduced in the double mutant, likely due to nonsense-mediated decay of these transcripts, while the expression of *ZmROS1c* and *ZmROS1d* were not changed (Additional file 1: Fig. S8b). This result is consistent with that using real-time qRT-PCR (Additional file 1: Fig. S2a). Previous study suggested that *ROS1* expression in maize may be positively regulated by ROS1 in a rheostat [32], we did not see such a rheostat in our mutants. However, both *zmros1a* and *zmros1b* are nonsense mutations, the nonsense-mediated decay could be a complicating factor in identifying such a rheostat. The comparison between WT and *zmros1ab* identified 2293 and 4957 differentially expressed genes in embryo and endosperm respectively (Additional file 1: Fig. S8c; Additional file 4). Both up- and downregulated genes were identified. When overlapping with DMRs, we found that there are more downregulated DEGs that have a hypermethylated DMR nearby than upregulated DEGs, especially in the CHG context (Additional file 1: Fig. S8d).

**Fig. 3:**
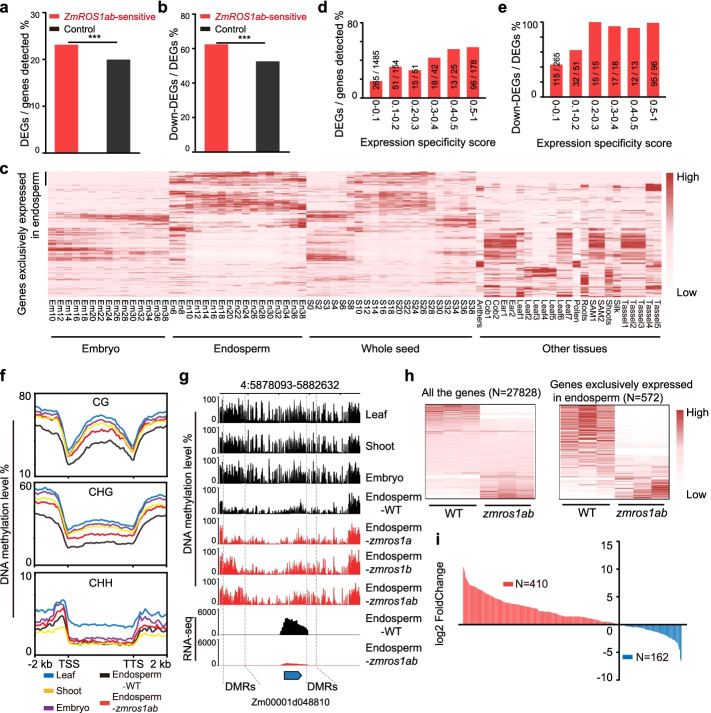
ZmROS1ab regulates gene expression in endosperm. **a** *ZmROS1ab*-sensitive genes are more likely to show differential expression in endosperm. **b** Most of the *ZmROS1ab*-sensitive DEGs show downregulation. ***, *P* < 0.001. **c** The tissue expression pattern of the *ZmROS1ab*-sensitive DEGs. The expression data was from Yi et al. [33]. Refer to Additional file 1: Fig. S1c for details of the tissues. **d** *ZmROS1ab*-sensitive genes with preferential expression in endosperm are more likely to show differential expression. The *x*-axis shows the degree of endosperm preferential expression from low (small values) to high (big values), and the *y*-axis shows the percentage of DEGs between WT and *zmros1ab* mutant. **e** *ZmROS1ab*-sensitive DEGs with preferential expression in endosperm are more likely to show downregulation. The *x*-axis is the same as **d**, and the *y*-axis is the percentage of downregulated genes in *zmros1ab* mutant compared to WT. **f** DNA methylation profile of *ZmROS1ab*-sensitive DEGs across various tissues and *zmros1ab* mutant. **g** An example to show DNA methylation levels in all three contexts and the expression level of a gene that is specifically expressed in endosperm. The position of the DMRs is indicated with dashed vertical lines. **h** Heatmap showing the expression levels of all expressed genes and endosperm-specific genes in WT and *zmros1ab* mutant. **i** The distribution of the fold change between WT and *zmros1ab*. The genes are ordered based on their fold change between WT and *zmros1ab*

We then tested whether the “*ZmROS1ab*-sensitive” DMRs in endosperm are more likely to associate with differential gene expression. These DMRs can be connected with 1983 expressed genes (within 2kb of genes), 459 (23%) of which are differentially expressed between *zmros1ab* and WT. This is significantly higher than the genome-wide average (4957/27828 = 17.8%, prop.test, *P* < 0.001, Fig. 3a). More than 60% of the “*ZmROS1ab*-sensitive” DEGs are downregulated in the *zmros1ab* mutant, which is higher than the genome-wide average (2608/4957=52.6%, prop.test, *P* < 0.001, Fig. 3b), suggesting that increased DNA methylation in the mutant is related to decreased gene expression. When examining the tissue-specific expression patterns of these 459 “*ZmROS1ab*-sensitive” DEGs, we found that 96 (21%) of them are preferentially expressed in endosperm, with 44 genes exclusively expressed in endosperm (see “Methods,” Fig. 3c). This prompted us to test whether there is any relationship between tissue specificity and differential gene expression. Interestingly, the percentage of genes showing differential expression, as well as the percentage of genes showing down expression increased as the degree of endosperm preference increased (Fig. 3d, e). This suggests that removal of DNA methylation by ZmROS1ab is required for the tissue-specific expression of these genes in endosperm. To further confirm this, DNA methylation levels across different tissues of these 459 genes were investigated. DNA methylation levels were reduced in endosperm compared to other tissues, and increased in *zmros1ab* endosperm (Fig. 3f; Additional file 1: Fig. S8e). An example is shown in Fig. 3g where a gene is exclusively expressed in endosperm. This gene has reduced DNA methylation only in endosperm and not in the other tissues. In the *zmros1ab* mutant, DNA methylation level was increased and gene expression level was reduced. A similar analysis was performed in embryo, which identified 29 CG DMRs and 43 CHG DMRs that are *ZmROS1ab*-sensitive DMRs in embryo (hypomethylated in embryo compared to endosperm and hypermethylated in *zmros1ab* embryo compared to WT embryo). These DMRs were associated with 13 genes, none of which is specifically expressed in embryo. This suggests that ZmROS1ab has a profound effect on gene expression in endosperm than in embryo.

To test broadly the role of ZmROS1ab on endosperm-specific expression, we identified 572 genes that are exclusively expressed in endosperm and looked at how their expression was affected in *zmros1ab* mutant. We found that the majority of these genes show a trend of downregulation in the *zmros1ab* mutant (Fig. 3h). Out of the 572 endosperm-specific genes, 410 showed decreased expression in *zmros1ab* mutant, with 177 (43%) reaching statistical significance (Fig. 3i). The other 162 endosperm-specific genes showed increased expression in *zmros1ab* mutant, with only 23 (14%) reaching significance level. Together, these results suggest that ZmROS1ab-mediated DNA demethylation is necessary for the tissue-specific expression of a subset of genes in endosperm.

### ZmROS1ab is required for imprinted expression of genes in endosperm

DNA methylation has been proposed to control imprinted expression in maize endosperm [26, 34, 35]. However, the direct role of ZmROS1ab-mediated DNA demethylation in maize imprinting has not been tested. As a first step towards answering this question, we asked whether previously described imprinted genes (Additional file 5) exhibit differential expression in the homozygous *zmros1ab* mutant compared to WT. Interestingly, 52 (25%) of the MEGs and 38 (27%) of PEGs are differentially expressed in *zmros1ab* mutant. This proportion is higher than the genome-wide control (17.8%, prop.test, *P* < 0.01 for both MEGs and PEGs), suggesting a role of ZmROS1ab in imprinting. When examining the direction of differential expression, we found that 73% of MEGs (38/52, prop.test, *P* < 0.01) showed downregulation, compared to a genome-wide average of 52.6% (2608/4957). There is also a minor preference for downregulation of PEGs but not reaching significance level (22/38=58%, prop.test, *P* > 0.05).

**Fig. 4:**
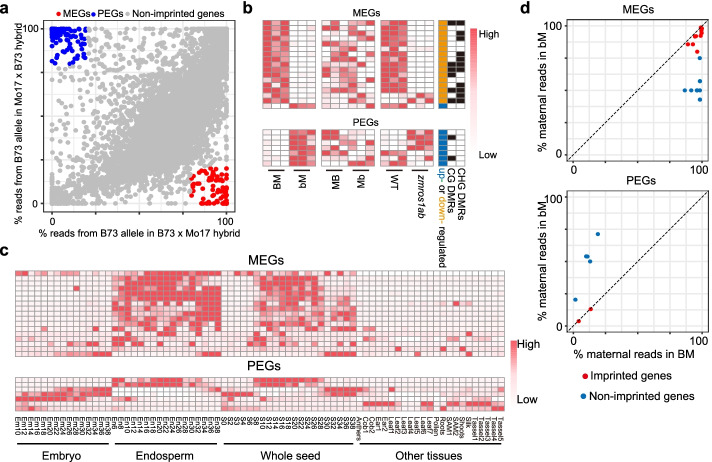
ZmROS1ab regulates imprinted expression in endosperm when inherited as the maternal allele. **a** Identification of imprinted genes in endosperm. **b** The expression levels of differentially expressed MEGs and PEGs between bM and BM F_1_ hybrid. The filled black boxes indicate presence of DMRs. The orange and blue boxes indicate down- or upregulation of these genes in bM relative to BM, respectively. **c** Tissue expression patterns of the MEGs and PEGs. The expression data was from Yi et al. [33]. Refer to Additional file 1: Fig. S1c for details of the tissues. **d** Comparison of the percentage of maternal reads (i.e., B73) in the BM and bM crosses

We hypothesized that ZmROS1ab is necessary for expression of the maternal allele for many MEGs. To test this hypothesis, we made crosses using either the WT or the *zmros1ab* mutant (both in B73 background) as the female parent and Mo17 as the male parent (hereafter referred as the BM for the WT cross and bM for the mutant cross). Comparison of gene expression between BM and bM allows us to test the effect of ZmROS1ab on the maternal allele. As a control, we also made reciprocal crosses using the WT (MB) or *zmros1ab* (Mb) as the male parent. Analysis of the two WT crosses BM and MB allowed the identification of 96 MEGs and 125 PEGs (Fig. 4). Among them, 34 MEGs and 86 PEGs were identified in previous studies (Additional file 1: Fig. S9a). Next, we identified differentially expressed genes between BM and bM (Fig. 4b). In total, 17 MEGs and 7 PEGs were differentially expressed. Tissue expression pattern analysis suggests that most of the differentially expressed MEGs were preferentially expressed in endosperm (hereafter “Endo-MEGs”), and most of the PEGs were constitutively expressed across different tissues (hereafter “Con-PEGs”, Fig. 4c). Interestingly, the majority of Endo-MEGs (16/17) show downregulation in bM cross, and all Con-PEGs (7/7) show upregulation in bM cross (Fig. 4b). Furthermore, 14 of the 17 Endo-MEGs show downregulation, and 4 of the 7 Con-PEGs show upregulation in the homozygous *zmros1ab* mutant compared to WT. This differential expression was not observed in the comparison of the reciprocal crosses (MB vs Mb), suggesting that the impact of ZmROS1ab on gene expression is exerted through the maternal allele (i.e., the central cell). To further test this idea, we analyzed expression of each allele by taking advantages of the SNPs between the parental lines B73 and Mo17. Interestingly, the expression levels of the maternal allele (B73) were changed between BM and bM, while no changes were observed on the paternal Mo17 allele (Additional file 1: Fig. S9b). More importantly, the proportion of the maternal reads for Endo-MEGs is significantly reduced in bM compared to BM, making 7 of the 17 Endo-MEGs no longer an imprinted MEG. Similarly, the proportion of the maternal reads for Con-PEGs is increased in bM compared to BM, making 5 of the 7 Con-PEGs no longer an imprinted gene in the bM cross (blue dots in Fig. 4d). Together, these results provide evidence that ZmROS1ab is required for the imprinted expression of some genes, especially some of the Endo-MEGs and Con-PEGs.

### Alteration in imprinted expression is accompanied by altered DNA methylation

To explore whether the differential expression between BM and bM in Endo-MEGs and Con-PEGs was accompanied by DNA methylation changes, we first checked whether there are DMRs around these genes in the homozygous mutant. Among the 17 Endo-MEGs, 12 have CG or CHG DMRs. Similarly, 2 of the 7 Con-PEGs have DMRs (Fig. 4b). Furthermore, a significant change in both CG and CHG contexts were observed in the gene body as well as in the 2-kb promoter region for the Endo-MEGs (Additional file 1: Fig. S9c). However, the methylation changes were only observed in the promoter region for the Con-PEGs (Additional file 1: Fig. S9d). Besides these differentially expressed Endo-MEGs and Con-PEGs between BM and bM, there are 9 other Endo-MEGs and 62 Con-PEGs that do not show differential expression. Analysis of DNA methylation levels across these imprinted genes also revealed changes in DNA methylation (Additional file 1: Fig. S9c, S9d), suggesting that expression of these genes are regulated by other factors as well.

**Fig. 5:**
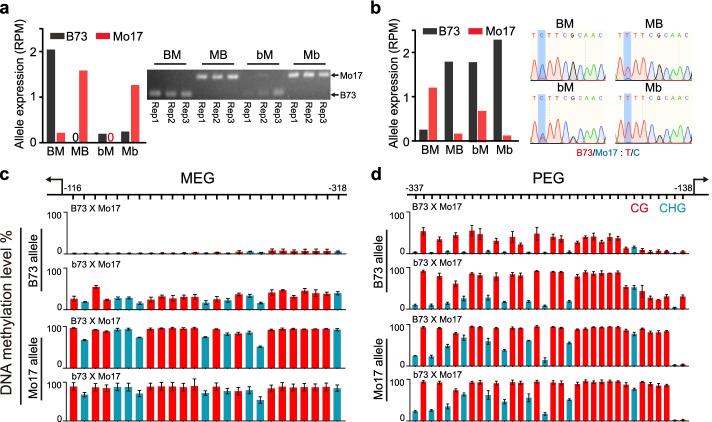
The imprinted expression in endosperm is related to DNA methylation. **a,b** Expression of the two alleles in either wild-type BM cross or in mutant bM cross of a MEG (**a**) and a PEG (**b**). The results from RT-PCR (right panel) and RNA-seq (bar plot in left panel) were shown. “0” means no reads were detected for the allele. **c,d** Methylation levels assayed using bisulfite PCR and sequencing for the MEG in **a** (**c**) and the PEG in **b** (**d**)

We hypothesized that ZmROS1ab is required for demethylation of the maternal allele and imprinted expression. To test this hypothesis, we performed bisulfite PCR and sequencing for one MEG (*Zm00001d024035*) and one PEG (*Zm00001d050109*) for which SNP is available to differentiate the two alleles (Fig. 5). We first confirmed with RT-PCR that these two genes are imprinted genes in the wild-type cross and that their expression pattern was altered in the *zmros1ab* mutant cross (Fig. 5a, b) For both genes, the maternal B73 allele was hypomethylated in the BM wild-type cross; however, methylation in both CG and CHG contexts increased in the bM cross (Fig. 5c, d). These results were further confirmed with Chop-PCR where a methylation-dependent enzyme (FspEI) digestion coupled with PCR was used (Additional file 1: Fig. S9e-g). These results are consistent with the idea that ZmROS1ab-mediated DNA demethylation is required to alleviate the repressed expression of Endo-MEGs. For the Con-PEGs, our results support the idea that demethylation of the maternal allele is required for deposition of other repressive marks (e.g., H3K27me3) on this allele to get silenced. Together, our analysis with the two examples provided evidence that allelic DNA methylation is linked to allelic gene expression of some imprinted genes, and ZmROS1ab-dependent DNA demethylation in central cell is critical for the expression of these imprinted genes.

### Increased DNA methylation in *zmros1ab* mutant is associated with reduced transcription factor binding

**Fig. 6:**
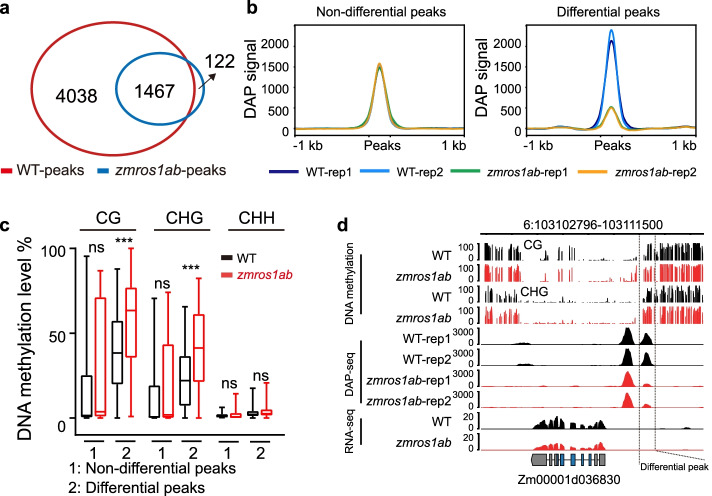
ZmROS1ab-mediated DNA demethylation affects transcription factor binding. **a** Overlaps of the ZmO2 binding peaks identified in WT and *zmros1ab* mutant. Peaks identified in both replicates were used for this analysis. **b** Identification of differential peaks between WT and mutant. **c** Comparison of DNA methylation levels in the differential and non-differential peaks. ***, *P* < 0.001; ns, not significant. **d** IGV view to show an example of differential binding of ZmO2 in WT and mutant

The above analyses suggest that ZmROS1ab regulates gene expression and imprinting of some genes in maize endosperm. To investigate the mechanisms of ZmROS1ab in regulating gene expression, we investigated the effect of DNA methylation on the binding of transcription factor to its target using DAP-seq. We chose ZmO2, a bZIP type transcription factor because it is a major regulator of gene expression in endosperm and has been successfully expressed in vitro. We asked whether the increased DNA methylation in *zmros1ab* mutant can affect the binding of ZmO2 to its target gene (Fig. 6; Additional file 1: Fig. S10). Two replicates were performed for both the WT and *zmros1ab* genotypes. The reproducibility of the two replicates is very high, as > 60% of the peaks can be identified in both replicates (Additional file 1: Fig. S10a, S10b). When considering the peaks that are identified in both replicates, 5505 and 1589 peaks were identified in the WT and *zmros1ab* mutant, respectively (Fig. 6a). The most highly enriched motif in these peaks is quite similar between the WT and the *zmros1ab* mutant, supporting high quality of the DAP-seq data (Additional file 1: Fig. S10c).

To study the relationship between DNA methylation and ZmO2 binding, we identified regions that show reduced binding by ZmO2 in the *zmros1ab* mutant compared to the WT. Regions with no difference in ZmO2 binding between WT and mutant were identified as controls (Fig. 6b). While there is no difference in DNA methylation in the control regions, significantly higher levels of DNA methylation was observed in *zmros1ab* mutant in the CG and CHG contexts for regions with reduced ZmO2 binding (Fig. 6c). As the enriched motif contains several cytosine sites (Additional file 1: Fig. S10c), we further checked whether the methylation status of this motif is associated with the differential binding strength. There is no difference in terms of the percentage of peaks containing this motif between the differential and non-differential peaks (Additional file 1: Fig. S10d). However, significant methylation difference within this motif was observed between WT and *zmros1ab* for the differential peaks (Additional file 1: Fig. S10e). The DAP-seq experiment was replicated on a separate day using a different biological replicate. As expected, the peak reproducibility between the two independent experiments is quite high (Additional file 1: Fig. S10f). The methylation difference in differential peak between WT and *zmros1ab* is reproducibly observed (Additional file 1: Fig. S10g, S10h). Besides, we used the PCR amplified DNA for DAP-seq. If the binding difference between WT and *zmros1ab* mutant was due to differential DNA methylation levels, the amplified samples would show no binding difference as PCR amplification removes DNA methylation. Indeed, the differential peaks between non-amplified WT and mutant are no longer showing difference (Additional file 1: Fig. S10i). Together, these results suggest that ZmO2 binding at some loci can be reduced by high DNA methylation levels.

Next, we examined whether the differential binding of transcription factors can lead to expression changes. Among the 98 differential peaks, 53 were associated with 107 expressed genes, of which 16 are differentially expressed between WT and *zmros1ab* mutant. One exemplar gene which shows increased DNA methylation, reduced ZmO2 binding and reduced expression levels was shown in Fig. 6d. Together, these results suggest that ZmROS1ab-mediated DNA demethylation can regulate expression of some genes possibly by affecting the binding strength of transcriptional factor to its targets.

## Discussion

### The hypomethylation in maize endosperm requires ZmROS1ab

Hypomethylation in maize endosperm relative to other tissues was initially observed at individual loci [34, 35]. Genome-wide analyses estimated that the endosperm exhibited a 13% reduction in total methylation compared to leaf based on HPLC [36]. We provided evidence that the hypomethylation in maize endosperm is partly due to active demethylation mediated by ZmROS1ab. In *zmros1ab* endosperm, DNA methylation was increased but was still lower than that in other tissues (Fig. 1). This is potentially because of the redundant roles of the other two genes *ZmROS1c* and *ZmROS1d*. Our results also suggest that the hypomethylation in endosperm most likely occurs on the maternal allele since the embryo which receives a similar sperm nucleus as the endosperm shows similar high levels of DNA methylation as the other tissues. Therefore, the methylation difference between embryo and endosperm (Fig. 2b) is most likely due to the difference between the egg cell and the central cell, as found in *Arabidopsis* and rice [9]. We should note that *ZmROS1a* is lowly expressed in the endosperm and that *ZmROS1a* and *ZmROS1b* are both highly expressed in embryo, the use of *zmros1ab* might inadvertently affect methylation levels of sporophyte tissues where gametophytes are derived from, and thus affecting *zmros1ab* endosperm methylome. It is also worth noting the potential for cryptic mutations from the EMS mutagenesis. Although we assessed the background mutations present in the mutant line based on genome-wide sequencing data (Methods), we cannot completely rule out the potential effect of any uncharacterized or unidentified mutations.

Why is the endosperm specifically being demethylated? Two possibilities are considered here. The first is that demethylation in endosperm/central cell leads to production of small RNA which can transfer to embryo/egg cell to reinforce a silencing state [37]. This possibility was supported by the tissue expression pattern of *DME* in *Arabidopsis* where it is preferentially expressed in the companion cells of both male and female gametophyte, thus avoiding subjecting the germline to potentially deleterious transposon demethylation [25, 37, 38]. A similar mechanism is likely present in maize. In support of this, we observed that many differentially expressed genes in *zmros1ab* mutant encode transposon-related functions (Additional file 6). A second possible reason for endosperm demethylation is facilitating the expression of genes that are specifically expressed in endosperm, particularly those that are involved in endosperm function. Indeed, among the DEGs in *zmros1ab* that also have a DMR, we found *Opaque2*, a master regulator of nutrient reservoir in endosperm (Additional file 6). Together, our results suggest a critical role of DNA demethylase in regulating DNA methylation levels and the biological function of the maize endosperm tissue.

In *Arabidopsis* and rice, loss of *DME* function leads to seed abortion [5, 14, 39]. Here we found that the kernels of the *zmros1ab* double mutants are viable, and the double mutant gamete was transmitted at the expected ratio in the self-pollinated ear of the *zmros1a*/+;*zmros1b*/+ plants. However, this does not necessarily mean that these two genes are not important. We noted that the homozygous double mutants of *zmros1ab* in general have smaller ears with poor seed-setting (Additional file 2: Fig. S2). And the kernel weight of the double mutant is smaller compared to WT. This suggests that the two genes are important for seed development. The lack of seed abortion is probably due to the redundant function of the other two genes, *ZmROS1c* and *ZmROS1d*. Further characterization of mutant alleles of these two genes would help to address this question.

### Regulation of imprinted expression in maize endosperm by DNA demethylation

Genome-wide surveys identified many imprinted genes where the two alleles are differentially methylated depending on the parent-of-origin [26, 40]. Based on the allelic methylation patterns in the hybrid endosperm, DNA demethylation was proposed to be involved in regulating imprinted expression in maize [22, 26]. However, a role of DNA demethylation in imprinting was not tested in maize. Here our results provide evidence that ZmROS1ab-mediated DNA demethylation is required for the imprinted expression of some genes. Specially, loss of *ZmROS1ab* leads to hypermethylation and reduced expression of the maternal allele of Endo-MEGs. This affect is likely occurring in the central cell because the differential expression of imprinted genes only occurs in the cross where the mutant allele is transmitted through the female gametophyte but not in the cross where the mutant allele is a pollen donor. This is also supported by their expression patterns where both *ZmROS1a* and *ZmROS1b* are highly expressed in ovule [41] and that *ZmROS1b* is highly expressed in endosperm (Additional file 1: Fig. S1c). This result is consistent with previous findings that DME-mediated DNA demethylation determine imprinted expression in *Arabidopsis* and rice [5, 27, 42] and suggest a conserved role of DNA demethylase in regulating imprinting.

Our results suggest that imprinted genes are not equally regulated by ZmROS1ab-mediated DNA demethylation. The Endo-MEGs and the Con-PEGs are more frequently affected than the other two types of imprinted genes: MEGs that are constitutively expressed (Con-MEGs) and PEGs that are specifically expressed in endosperm (Endo-PEGs). This suggests the involvement of other mechanisms, such as histone modifications in regulating imprinting [22, 26, 43]. Besides, not all Endo-MEGs and Con-PEGs identified are affected by ZmROS1ab, suggesting a redundant role of the other two genes encoding DNA demethylase in maize. Nevertheless, our results confirmed that removal of DNA methylation mediated by DNA demethylase in the central cell is required for the imprinted expression of genes in maize endosperm.

### DNA methylation may affect gene expression via regulating protein binding to its target

It was reported that DNA methylation levels are usually negatively associated with gene expression levels at the genome-wide scale [44]. Similarly, many epialleles where hypermethylation leads to lower gene expression or vice versa have been reported [45–47]. However, how high DNA methylation levels can inhibit gene expression is largely unexploited. One possibility is that the DNA methylation found within the binding motif can decrease protein binding. In rice, the binding of RELATIVE OF EARLY FLOWERING 6 (REF6/JMJ12), a H3K27me3 demethylase with a zinc-finger domain that can recognize the CTCTGYTY motif, was greatly reduced by CHG methylation within the binding motif [48]. In *Arabidopsis*, a high-throughput survey of the binding sites of hundreds of transcription factors found that > 75% of the transcription factors show methylation-sensitive binding [49]. In maize, it was shown at individual loci that the presence of a single methylated cytosine within the core binding motif can diminish the affinity of transcription factor for the target sequence [50]. Here, we showed at a genome-wide scale that higher DNA methylation levels are associated with reduced binding of a transcription factor to its target sites and reduced gene expression (Fig. 6). This can probably explain why some genes show tissue-specific expression, as found here for the genes that are exclusively expressed in endosperm (Fig. 3). It is possible that the DNA methylation levels are variable across different tissues, and the gene can only be expressed at the tissue where DNA methylation was reduced, and thus facilitating the access of gene regulators.

While our data suggests a possible inhibitory effect of DNA methylation on the binding of transcriptional regulators, this does not necessarily mean DNA methylation always lead to reduced gene expression since reduction in binding of repressors can in fact lead to increased gene expression. Besides, there are also cases where DNA methylation can facilitate regulator binding leading to increased gene expression [51–55]. These could probably explain why both up- and downregulation were observed when DNA methylation levels are perturbed. Together, these suggest a diverse role of DNA methylation in gene expression via regulating the binding of transcriptional regulators to their target sites.

## Conclusions

In conclusion, the occurrence and function of ZmROS1ab-mediated DNA demethylation was investigated in maize. We showed that ZmROS1ab is required to establish the methylation status in endosperm, which shows genome-wide hypomethylation compared to other tissues. Loss of *ZmROS1ab* function leads to methylation changes preferentially around genes. The methylation changes in *zmros1ab* mutant can affect transcription factor binding and are critical for the proper expression of genes in endosperm, including endosperm-specific genes and imprinted genes. These findings extend the role of DNA demethylation to an important crop species and provide a mechanistic insight into gene expression regulatory by DNA demethylation.

## Methods

### Materials used in this study

To identify maize genes encoding DNA demethylase, the *Arabidopsis* genes encoding DNA demethylase were used to blast against gene annotations from *Zea mays* AGPv4. Four genes *Zm00001d038302* (*ZmROS1a*), *Zm00001d053251* (*ZmROS1b*), *Zm00001d016521* (*ZmROS1c*), and *Zm00001d016516* (*ZmROS1d*) were identified with an E-value of less than 1E−10. There was an annotation error of *ZmROS1d* in the reference genome where some amino acids from the C-terminal were missed. We corrected this error based on our RNA-seq data. The maize ROS1 protein sequences were used to blast the genomes of tomato and rice to obtain its homologs. The phylogenetic tree was inferred using the full-length protein sequences from maize, *Arabidopsis*, rice, and tomato using the maximum likelihood method in MEGA 7.0.

Two single mutants for *ZmROS1a* and *ZmROS1b* were identified by Lu et al. [56] in a large population of sequence-indexed mutations resulting from EMS mutagenesis, and these mutants can be obtained through the website http://elabcaas.cn/memd/. Both mutants were in the B73 genetic background. Cleaved amplified polymorphic sequence (CAPS) markers were developed for each mutant for genotyping (Additional file 1: Table S2). The double mutant was obtained by crossing the two single mutants, and the two CAPS markers were used to select the double mutants. The mutants, their WT control, and the inbred line Mo17 were planted in an experimental field in Sanya of Hainan province, China. Embryo and endosperm of kernels 14 days after self-pollination were collected from the WT, single and double mutants, as well as the reciprocal F_1_ hybrids between the WT (or double mutant) and Mo17. The samples were frozen in liquid nitrogen immediately and stored in – 80 °C until use.

For the samples used for WGBS, RNA-seq, DAP-seq, and qRT-PCR, the samples were obtained as follows. The WT and *zmros1a* mutant samples were generated by self-pollination of a heterozygous plant to produce WT and homozygous mutant individuals. These WT and mutant plants were then self-pollinated for two generations prior to sampling for the WT and *zmros1a* samples. The *zmros1b* sample was isolated from a plant that had been maintained as homozygous mutant for 5 generations prior to sampling. The *zmros1ab* double mutant was generated by crossing single mutants of *zmros1a* and *zmros1b* following by self-pollination of the resulting double heterozygous plants. The isolated double mutant was then self-pollinated for two generations prior to sampling. These samples were used for WGBS, RNA-seq, and qRT-PCR. The DAP-seq samples were propagated the same but with one more generation of self-pollination.

For the phenotyping and imprinting analysis (including RNA-seq, Chop-PCR, and bisulfite PCR), the two single mutants were crossed to get the double heterozygotes, which were then self-pollinated to obtain the homozygous WT, single mutants, and the double mutant. Phenotypic analysis was performed after self-pollination of each of the homozygous stocks. Each of these homozygous stocks was self-pollinated for two generations to confirm the genotypes and was then crossed with Mo17 as both male and female and the kernels were sampled for RNA-seq, bisulfite PCR, and Chop-PCR analysis.

For the analysis of transmission of the single mutants, each single mutant was backcrossed twice (*zmros1a*) or three times (*zmros1b*) to B73, and the heterozygous plants were self-pollinated. The resulting kernels were genotyped using endosperm tissue. For the transmission of the double mutant, each single mutant was backcrossed to B73 four times (*zmros1a*) or five times (*zmros1b*) and was then crossed to each other to generate double heterozygotes, which were self-pollinated. The endosperm of the kernels from the self-pollinated ears was genotyped using the two CAPS markers (Additional file 1: Table S2).

### Phenotyping

Several traits were phenotyped for both the WT and the mutants, including plant height, ear height, flowering time, and hundred kernel weight. For plant height, ear height and flowering time, at least 10 plants were used for each genotype. For hundred kernel weight, at least 50 kernels from the middle part of at least 7 ears were used and the average was used to represent the value of a particular genotype.

### Library preparation and sequencing

The standard CTAB method was used to prepare DNA for whole-genome bisulfite sequencing (WGBS). Libraries were constructed using 1 μg of genomic DNA based on a previously published protocol [57]. Library quality was checked using Qubit and gel electrophoresis. Sequencing was performed on HiseqX under the paired-end mode (2 × 150 bp) by ANOROAD (China).

For RNA-seq to identify differentially expressed genes (DEGs) between WT and *zmros1ab* mutant, three biological replicates from the embryo and endosperm of each genotype were used. Total RNA was extracted using Quick RNA isolation Kit (Hua yue yang, ZH120). The RNA libraries were prepared and sequenced at BGI (China) using BGISEQ-500. The adapters and low-quality (Q< 20) nucleotides were trimmed to get clean data.

For RNA-seq of F_1_ hybrids, the endosperm from reciprocal crosses between either WT or *zmros1ab* mutant and Mo17 was used. Three replicates were prepared for each cross. The inclusion of biological replicates allows us to identify DEGs between the WT and mutant crosses. Meanwhile, it allows us to identify imprinted genes when pooling these three replicates together. Total RNA was extracted by Trizol (Thermo Fisher Scientific). The RNA libraries were prepared and sequenced at ANOROAD (China) using Illumina NovaSeq 6000.

For DAP-seq and ampDAP-seq (similar as DAP-seq but DNA methylation was removed by PCR amplification) of ZmO2, the full-length coding sequences of ZmO2 were cloned into the pFN19K HaloTag vector to make a HALO-tagged ZmO2. A HALO-tagged GFP vector was constructed similarly and separately as a control. The ZmO2 and GFP proteins were expressed in vitro using TNT SP6 Wheat Germ Master Mix (Promega L3261) following the manufacturer’s instruction. Separately, DNA from WT and *zmros1ab* mutant endosperm was prepared by the similar method used for WGBS. A total of 10 μg genomic DNA was fragmented to ~200 bp and selectively recovered by magnetic beads (KAPA Pure Beads, KK8002). Adapters were ligated using NEXTflex Rapid DNA-Seq Kit (PerkinElmer 5144-08). Next, the native adapter-ligated DNA was incubated with the HALO-tagged ZmO2 or GFP (input control) protein for DAP-seq experiment. As for ampDAP-seq, we used a PCR-amplified genomic DNA library to remove DNA methylation from the native DAP-seq DNA library for protein binding. The native or the amplified DNA bound by ZmO2 or GFP was eluted and PCR amplified using KAPA HiFi HotStart ReadyMix PCR Kit (Roche, KK2602). After purification, the libraries were sequenced on Illumina NovaSeq 6000 under the paired-end mode (2 × 150 bp) by ANOROAD (China). All experiments were carried out in two independent technical replicates.

### Analysis of WGBS data

The WGBS data was generated either in this study (PRJNA739488) or downloaded from NCBI (PRJNA529106 for leaf and shoot) [58]. After getting the raw reads, adapters were trimmed using Trim_Galore (http://www.bioinformatics.babraham.ac.uk/projects/trim_galore/), low-quality (Q < 20) reads were discarded. The remaining reads were mapped to the *Zea mays* AGPv4 reference genome (B73) using BSMAP allowing up to five mismatches [59]. Reads that are mapped uniquely were kept. Duplicate reads and reads that are not properly paired were removed using Picard Tools (https://broadinstitute.github.io/picard/) and Bam Tools [60]. The methylation levels at each individual cytosine were called using methratio.py in BSMAP. Reads that were mapped to the Chloroplast genome were used to calculate the conversion rate of bisulfite treatment (Additional file 2).

### Identification of DMRs

To identify DMRs, the maize genome was first divided into consecutive non-overlapping 100-bp windows. Next, the number of cytosine sites and the average coverage for each sequence contexts (CG/CHG/CHH) within each window were calculated for each sample. The 100-bp windows that have at least 6 cytosine sites and at least 3× coverage were retained. The average DNA methylation level of these remaining windows was calculated using the weighted DNA methylation computing method [61]. Windows were kept if the difference of DNA methylation level between WT and mutants was ≥ 30% for CG and CHG and ≥ 10% for CHH. These windows were considered as putative DMRs and were subject to following filtering: DNA methylation difference at each cytosine was calculated for each window between the contrast genotypes, and the 100-bp windows were removed if the percentage of differentially methylated cytosines (defined as ≥ 20% for CG/CHG, ≥ 10% for CHH) was less than 80%. Finally, the adjacent windows within 100 bp of each other were merged and DNA methylation levels of these merged regions were calculated. These regions were subject to further filtering based on their size. For regions that are 100 bp, we required the methylation difference is ≥ 60% for CG, ≥ 50% for CHG, and ≥ 20% for CHH. For DMRs that are > 100 bp, we required the methylation difference is ≥ 30% for CG, ≥ 30% for CHG, and ≥ 10% for CHH.

### Meta-analysis of DNA methylation levels

For metaplots of DNA methylation levels around the genes and TE, the maize genome was first divided into consecutive non-overlapping 100-bp windows. The average DNA methylation level of the 100-bp windows was calculated by the weighted DNA methylation computing method [61]. Next, the windows were linked with the nearest genes (Zea mays AGPv4) or TEs (https://mcstitzer.github.io/maize_TEs/) using Bedtools [62]. The 100-bp windows that are within 2 kb of the interested feature were kept. The upstream 2 kb and downstream 2 kb were divided into 20 equal bins each with a size of 100 bp. The length of the gene/transposon body was normalized to 4000 bp by dividing the distance between the 100-bp windows and transcriptional start site (TSS) to the size of the gene/transposon, multiplied by 4000. The gene/transposon body was divided into 40 equal bins. Methylation at CG, CHG, and CHH was calculated for each bin for each feature (gene/transposon), and the average DNA methylation levels were plotted against the position of each bin.

### Analysis of RNA-seq data

Raw data were trimmed using Trim_Galore, and clean reads were mapped to the *Zea mays* AGPv4 reference genome using Tophat2 with the parameters -g 5 [63]. Only uniquely mapped reads were retained. To obtain gene expression levels, the read counts per gene model were collected using the htseq-count command of HTSeq [64]. In total, 28,153–27,828 genes were detected to be expressed in embryo and endosperm. DEGs between WT and *zmros1ab* mutant were identified using the DESeq2 with the criteria: log_2_ fold change was ≥ 0.5 or ≤ − 0.5, and the adjusted *p* value ≤ 0.01. PCA was analyzed by plotPCA from DESeq2 [65].

As the mutants were generated using EMS mutagenesis and may contain many background mutations, the RNA-seq data was used to identify mutations compared to the reference B73 genome. The three biological replicates of each sample were pooled together to call SNPs using GATK HaplotypeCaller of Genome Analysis Toolkit (GATK, v3.7) with “--filter_reads_with_N_cigar” [66]. Next, SNPs were filtered out using the GATK VariantFiltration with the following parameters “QUAL < 30.0; QD < 2.0; MQ < 40.0; FS > 40.0; haplotype score > 13.0; MQRankSum < –12.5; ReadPosRankSum < –4.0”. The remaining SNPs covered by more than thirty reads were kept as confident SNPs. In total, we identified 576 non-redundant homozygous SNPs in the four samples (embryo and endosperm of WT and *zmros1ab* mutant) located within 364 genes. Among these genes, six has a putative annotation related to epigenetic regulation. Two of the genes are *ZmROS1a* and *ZmROS1b*. The other four genes are *Zm00001d035228*, *Zm00001d016340*, *Zm00001d021910*, and *Zm00001d011490*. The SNP within *Zm00001d035228* is the same between the WT and the *zmros1ab* mutant, while the SNPs within the other three are polymorphic between WT and the *zmros1ab* mutant. The *Zm00001d016340* gene has a SNP that is not within the coding region. The *Zm00001d021910* has five SNPs with one being a non-synonymous mutation (Thr to Ala), but this gene is undetectable in seed, endosperm, and embryo (FPKM < 0.1). The *Zm00001d011490* gene also has a non-synonymous mutation (Glu to Lys). This mutation is not within protein domains and the close homolog of this gene in maize genome (*Zm00001d008941*) has a corresponding Ser at the mutated position. Also this amino acid is not conserved in *Arabidopsis* and rice. The WGBS data was used to identify SNPs within the four *ZmROS1* homologs and only the expected SNPs in *ZmROS1a* and *ZmROS1b* were identified, no additional mutations were found in *ZmROS1c* and *ZmROS1d*. Based on all of this, we concluded that there are many background mutations in the mutant, but these mutations are not likely to cause changes in DNA methylation.

### Analysis of DAP-seq data

ZmO2 DAP-seq and ampDAP-seq data was trimmed using Trim_Galore. The remaining reads were mapped to maize AGPv4 reference genome using Bowtie2 with the parameters “--no-mixed --no-discordant” [67]. Next, duplicate reads were removed using Picard Tools and multiply mapped reads were removed using SAMTools [68]. MACS2 was used to define ZmO2 peaks with “-f BAMPE -g 2.1e9 -q 0.00001” for each of the two replicates separately [69]. DiffBind R package was used to define differential peaks (*q* ≤ 0.0001) and non-differential peaks (*q* ≥ 0.6, fold ≤ 0.001) with the DBA_DESEQ2 method (http://bioconductor.org/packages/release/bioc/vignettes/DiffBind). To identify the shared peaks between the two replicates, the peaks from the two replicates were intersected and the size of the overlapping regions was summarized. If the size of the overlapping region is > 90% of any one peak from the two replicates, the two peaks are regarded as shared and the overlapping region was used to represent the peak for the respective genotype. The shared peaks were used to identify binding motifs using MEME-ChIP [70].

### Identification of genes that are specifically expressed in endosperm

To identify the genes that are specifically expressed in endosperm, we collected gene expression levels from different tissues from the published data [33]. The gene expression levels in embryo, endosperm, and the whole seed were collected from different stages, and the maximum expression levels across all stages was used to represent expression levels in the respective tissue. The expression level in endosperm was divided by the total expression in all tissues, and if this ratio is higher than 50%, a gene was classified as preferentially expressed in endosperm. For genes that are exclusively expressed in endosperm, this ratio was required to be at least 90%. For genes that are not specifically in endosperm, this ratio was required to be less than 30%.

### Analysis on imprinting

To identify the imprinted genes, we obtained SNPs between B73 and Mo17 from the Data Repository for University of Minnesota (DRUM, 10.13020/D6T38X). Next, we made Mo17-like genome by converting the B73 allele to Mo17 allele at the SNPs on the basis of the B73 genome. The raw RNA-seq data of the three biological replicates were merged, which were then mapped to both the B73 genome and the Mo17-like genome using Tophat2 with the parameters “-g 5 --read-mismatches 0 --read-gap-length 0 --read-edit-dist 0 --no-discordant --no-mixed”. The uniquely mapped reads were retained for following analysis. To obtain allelic expression, the reads that were mapped to both the B73 genome and the Mo17-like genome were removed and the remaining reads were used to count allelic reads by the htseq-count command of HTSeq. The imprinted genes were then identified by the following criteria. First, genes that have at least 10 reads for one allele in both reciprocal crosses between the WT and Mo17 were retained. Next, genes with ≥ 80% of the reads coming from the maternal (paternal) parents in both reciprocal hybrids were identified as potential MEGs (PEGs). There is a possibility that these potential MEGs were due to contamination by maternal tissues. Therefore, a subset of the MEGs exhibited at least threefold higher expression in whole seeds relative to the endosperm tissue was removed, and the remaining subset was classified as MEGs. Previously identified imprinted genes were collected from published data [19–22].

To measure DNA methylation levels of the imprinted genes using bisulfite PCR and sequencing, the genomic DNA was treated with EZ DNA Methylation-Lightning Kit (Zymo, D5031) following the manufacturers’ instructions. The treated DNA was PCR amplified using KAPA HiFi HotStart Uracil+ReadyMix (KK2801) and the primers in Table S2. The PCR procedures were as follows: 95 °C for 5min followed by 35 cycles of “95 °C for 30 s, 55 °C for 30 s, 72 °C for 30 s” and a final extension at 72 °C for 5 min. The PCR product library was prepared and sequenced at ANOROAD (China) using Illumina NovaSeq 6000. The raw sequencing data was analyzed using a method similar to that used for WGBS data, except that allele-specific methylation levels were calculated. Specifically, the sequencing reads were differentiated according to SNPs that are present between the two parents B73 and Mo17. Allele-specific methylation levels were then summed using reads assigned to each allele.

## Supplementary Information

**Additional file 1.** Figure S1-S11 and Table S1-S2.

**Additional file 2.** Summary of data generated in this study.

**Additional file 3. **List of DMRs between *zmros1* and WT.

**Additional file 4. **List of DEGs between *zmros1* and WT.

**Additional file 5.** Imprinted genes from literatures and this study.

**Additional file 6. **List of genes that are related with *ZmROS1ab*-sensitive DMRs.

**Additional file 7.** Review history.

## Data Availability

All the data generated in this study was deposited in NCBI under project number PRJNA739488 https://www.ncbi.nlm.nih.gov/bioproject/PRJNA739488 [71]. The WGBS data for leaf and shoot (PRJNA529106, https://www.ncbi.nlm.nih.gov/geo/query/acc.cgi?acc=GSE128859) was from a previous study [58].
