# Supplementary material for: DNA demethylation affects imprinted gene expression in maize endosperm
Source: Genome Biol. 2022 Mar 9;23:77. doi: 10.1186/s13059-022-02641-x (PMC8905802; doi:10.1186/s13059-022-02641-x)
Supplement: Supplementary file 1 — Additional file 1. Figure S1-S11 and Table S1-S2. [file 13059_2022_2641_MOESM1_ESM.pdf]

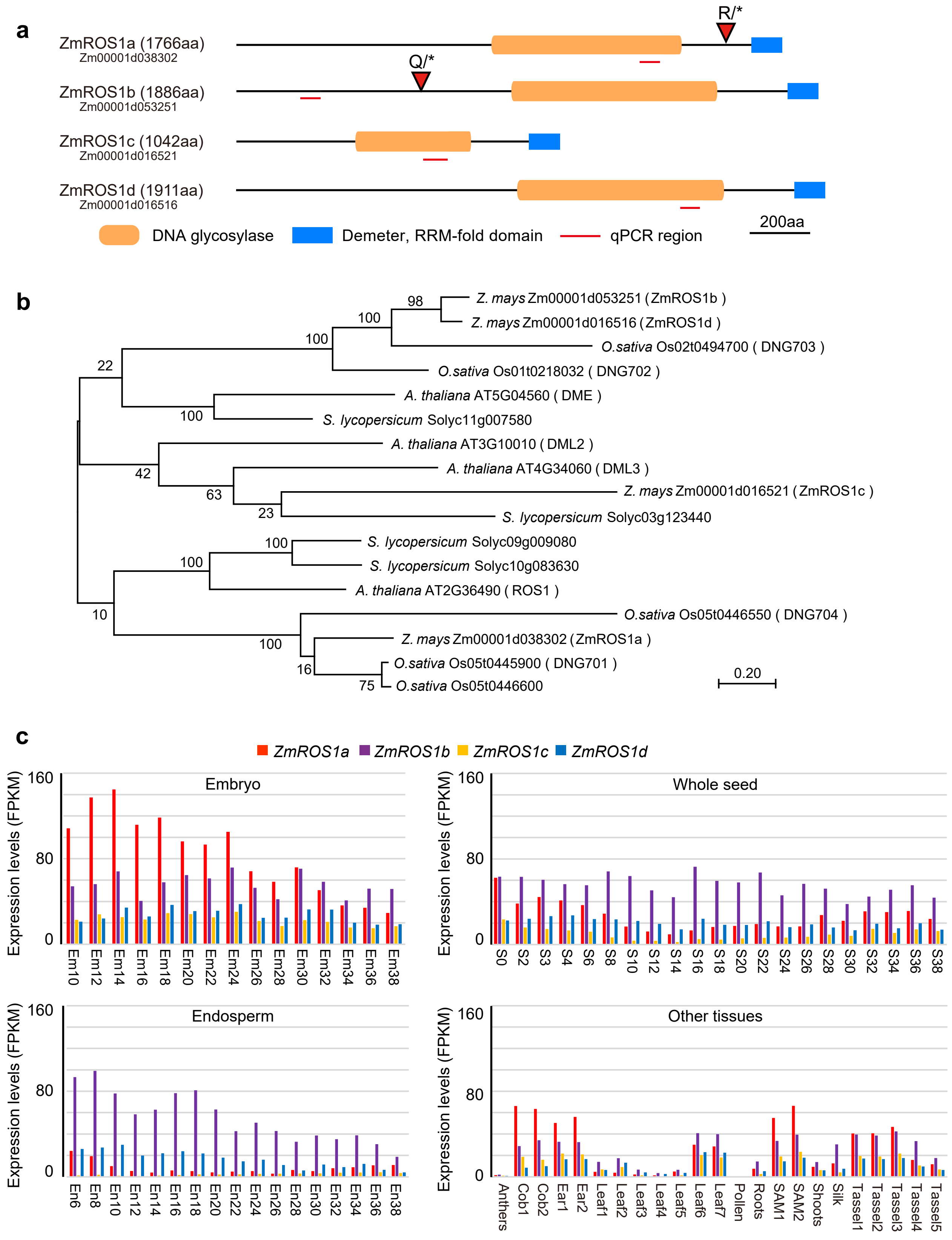

Figure S1 Genes encoding DNA demethylase in maize. (**a**) Protein domains of maize ROS1 genes. (**b**) Phylogenetic analysis of ROS1 genes from maize, *Arabidopsis*, rice and tomato. The numbers next to the branches show percentage of trees in which the associated taxa clustered together. The branch lengths were measured in the number of substitutions per site. (**c**) Expression pattern of maize ROS1 genes across tissues and developmental stages. The expression data was from published data [33]. Embryos (10 - 38 days after pollination, DAP) and endosperms (6 - 38 DAP) were sampled every 2 days. Whole seeds were sampled from 0 DAP to 38 DAP every 2 days. The bars were arranged according to the developmental stages. Other tissues (from left to right) include anther, cob (2 samples), ear (2 samples), leaf (7 samples), pollen, root, SAM (2 samples), shoot, silk and tassel (5 samples).

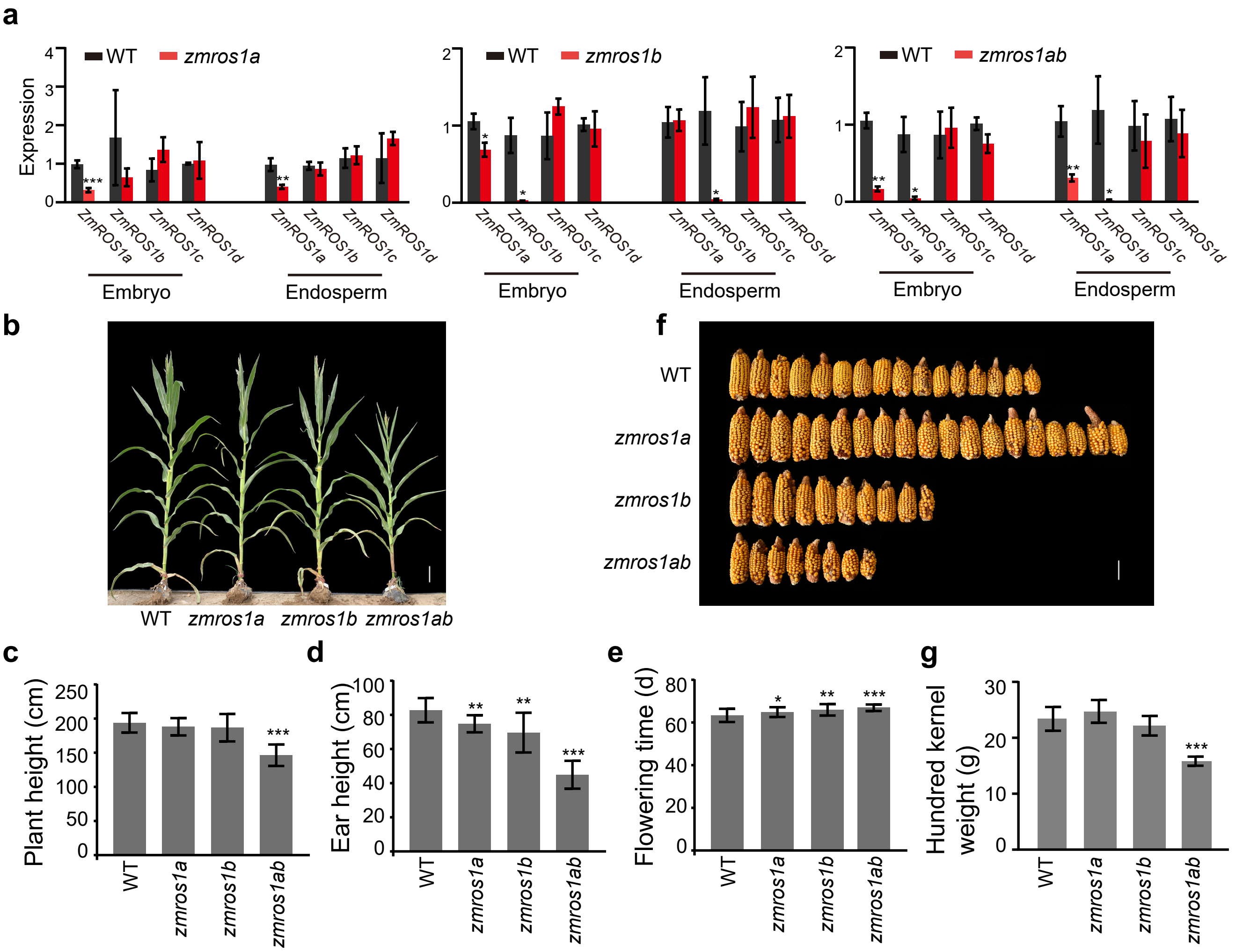

h

+/zmros1a

Genotype	Observation	Expectation
+/+	18	23.75
+/zmros1a	54	47.50
zmros1a	23	23.75
P	0.316	

i

+/zmros1b

Genotype	Observation	Expectation
+/+	29	24.00
+/zmros1b	48	48.00
zmros1b	19	24.00
P	0.353	

j

+/zmros1a;+/zmros1b

Genotype	Observation	Expectation
+/+;+/+	13	11.88
+/+;+/zmros1b	21	23.75
+/+;zmros1b	12	11.88
+/zmros1a;+/+	22	23.75
+/zmros1a;+/zmros1b	37	47.50
+/zmros1a;zmros1b	31	23.75
zmros1a;+/+	15	11.88
zmros1a;+/zmros1b	25	23.75
zmros1a;zmros1b	14	11.88
P	0.607	

Figure S2 Characterization of *zmros1* mutants. (**a**) Expression levels of the four *ZmROS1* genes in single and double mutants of *ZmROS1a* and *ZmROS1b*. *, P<0.05; **, P<0.01; ***, P<0.001. The significance level was based on student's t-test of three replicates of qRT-PCR data. Actin was used as control. Refer to **Figure S1** (**a**) for primer positions. (**b**) Representative plants of WT and mutants. Scale bar is 15 cm. (**c-e**) Bar plot to show plant height (**c**), ear height (**d**) and flowering time (**e**) of WT and mutants. (**f**) Representative ears of WT and mutants. Scale bar is 4 cm. (**g**) Hundred kernel weight of WT and mutants. (**h-j**) Transmission of the single or double mutant alleles of *zmros1a* and *zmros1b*. The P value is from chi-square test.

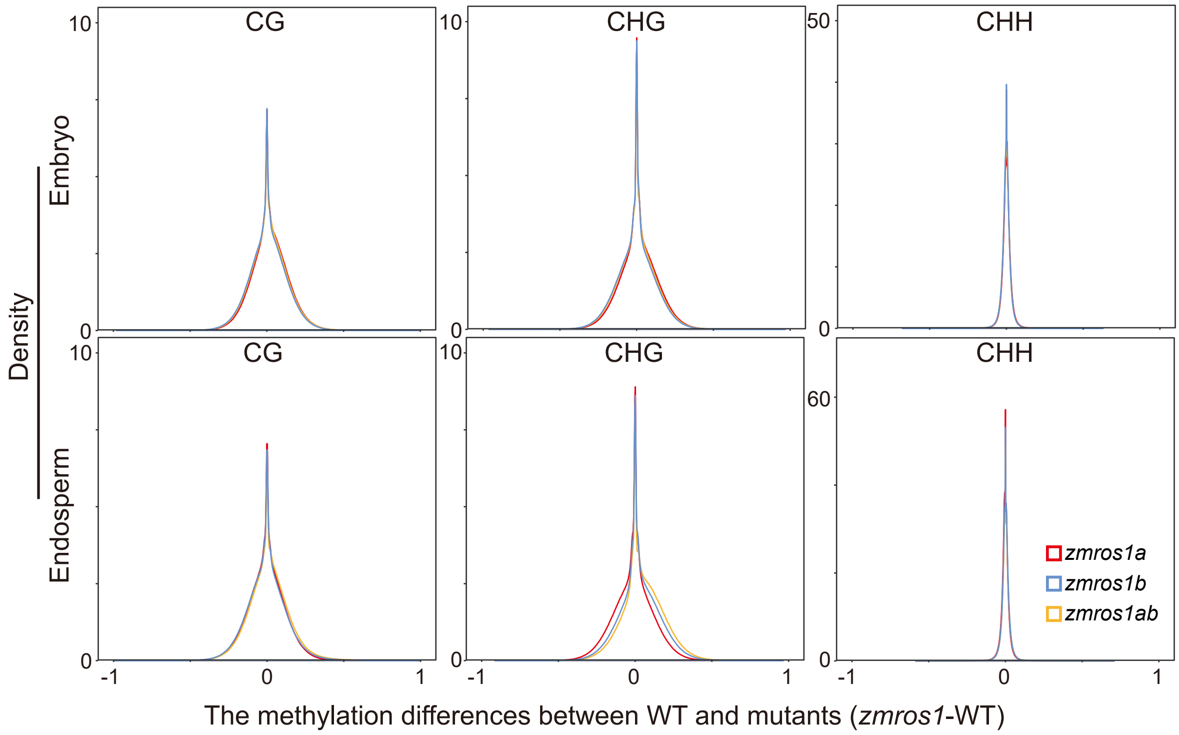

Figure S3 Global methylation changes in *zmros1* mutants. The six density plots show methylation changes in CG/CHG/CHH contexts in embryo and endosperm. The x axis is the methylation differences between WT and mutants and the y axis is the density. The 100-bp tiles that have enough coverage (3X) were used to perform this analysis.

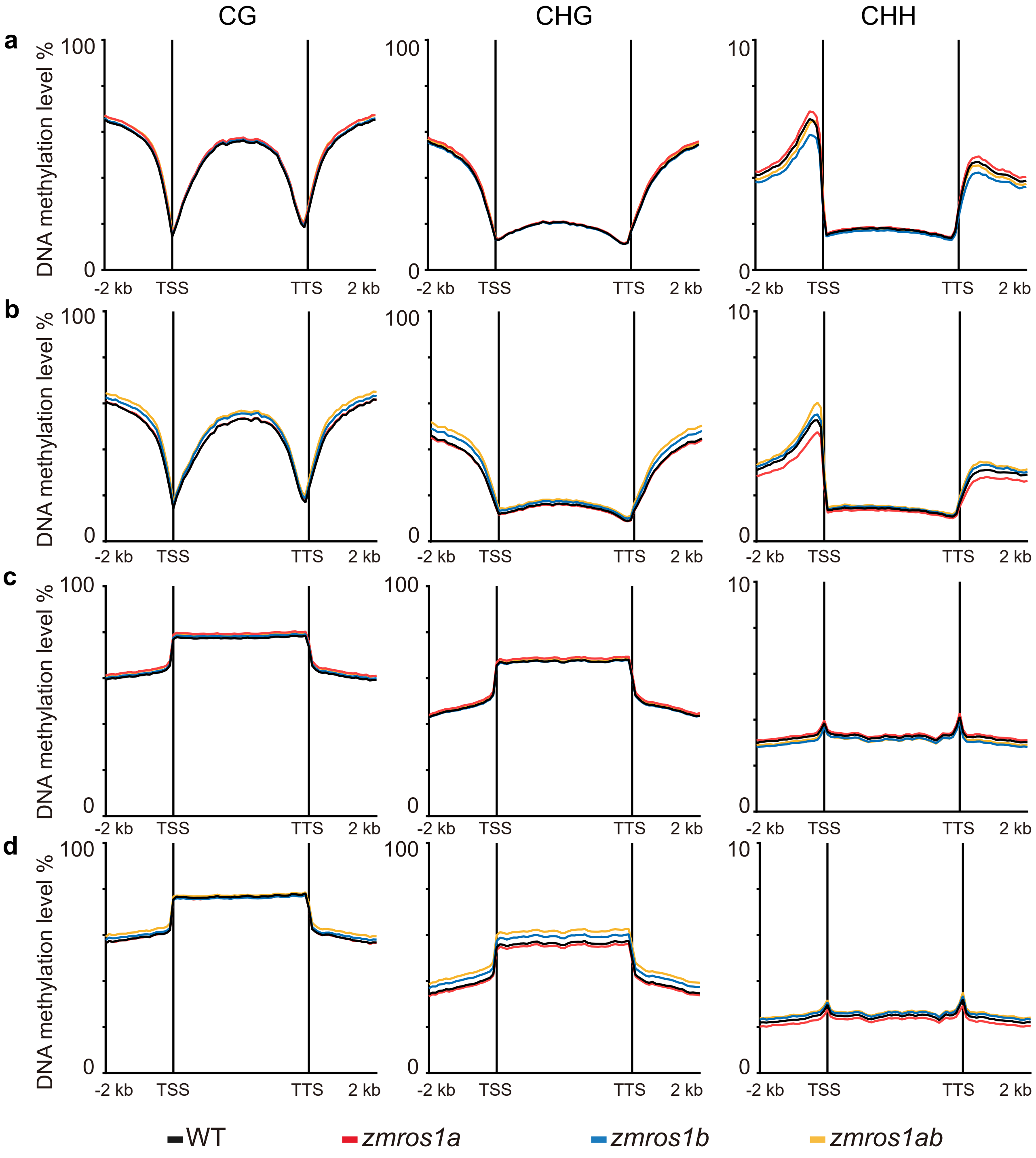

Figure S4 Patterns of DNA methylation in embryo and endosperm of WT and *zmros1* mutants across genes and transposons. (**a**) DNA methylation levels surrounding genes in WT and mutants for CG, CHG and CHH in embryo. (**b**) Similar as (**a**) but shows DNA methylation levels in endosperm. (**c-d**) DNA methylation levels surrounding transposons in embryo (**c**) and endosperm (**d**).

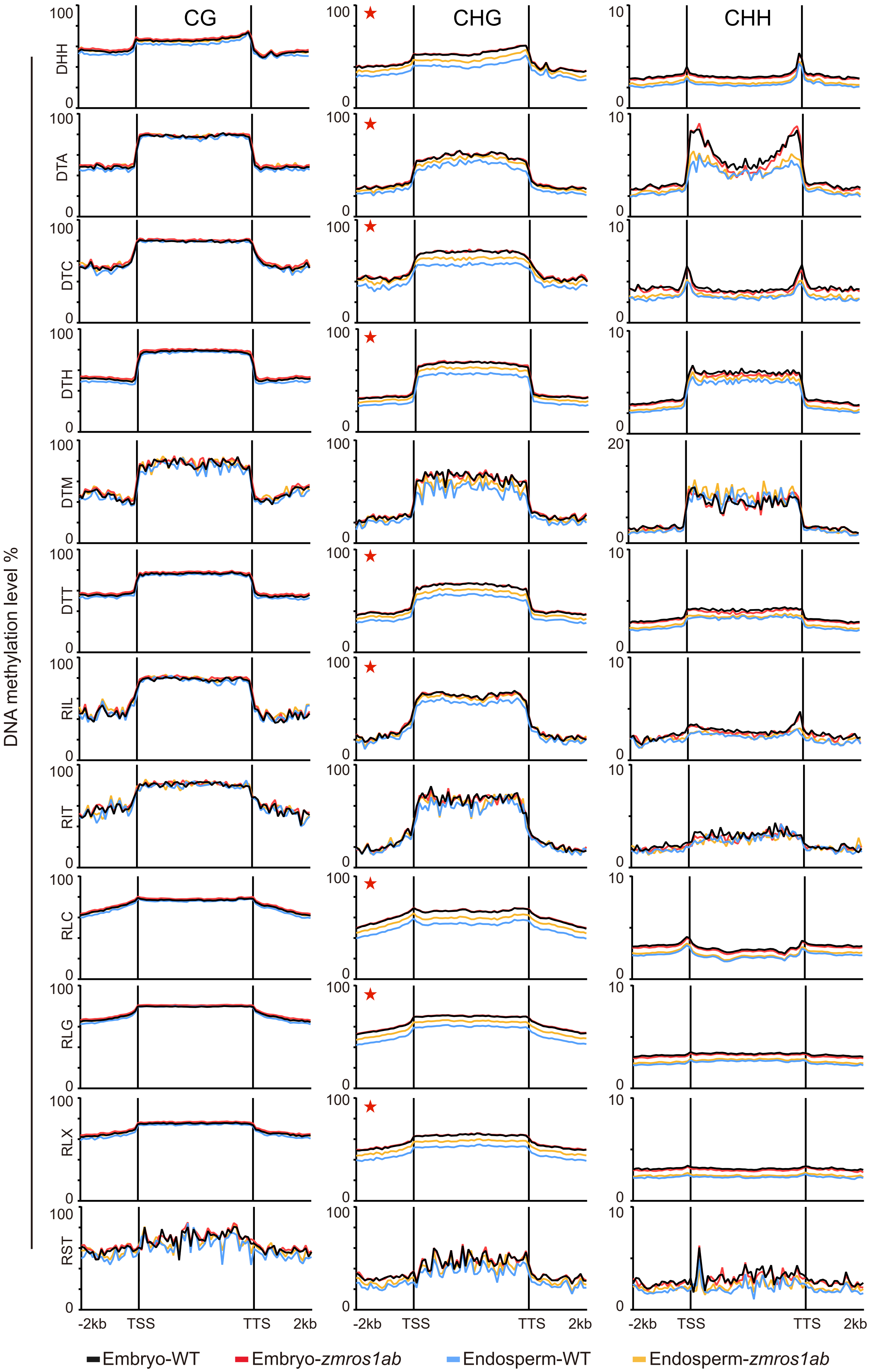

Figure S5 DNA methylation patterns of individual TE families in WT and *zmros1ab* double mutant in embryo and endosperm. Note that the plots with obvious DNA methylation changes between WT and mutant were labeled with red stars. The CHG context shows the most obvious changes, while CG and CHH show little changes.

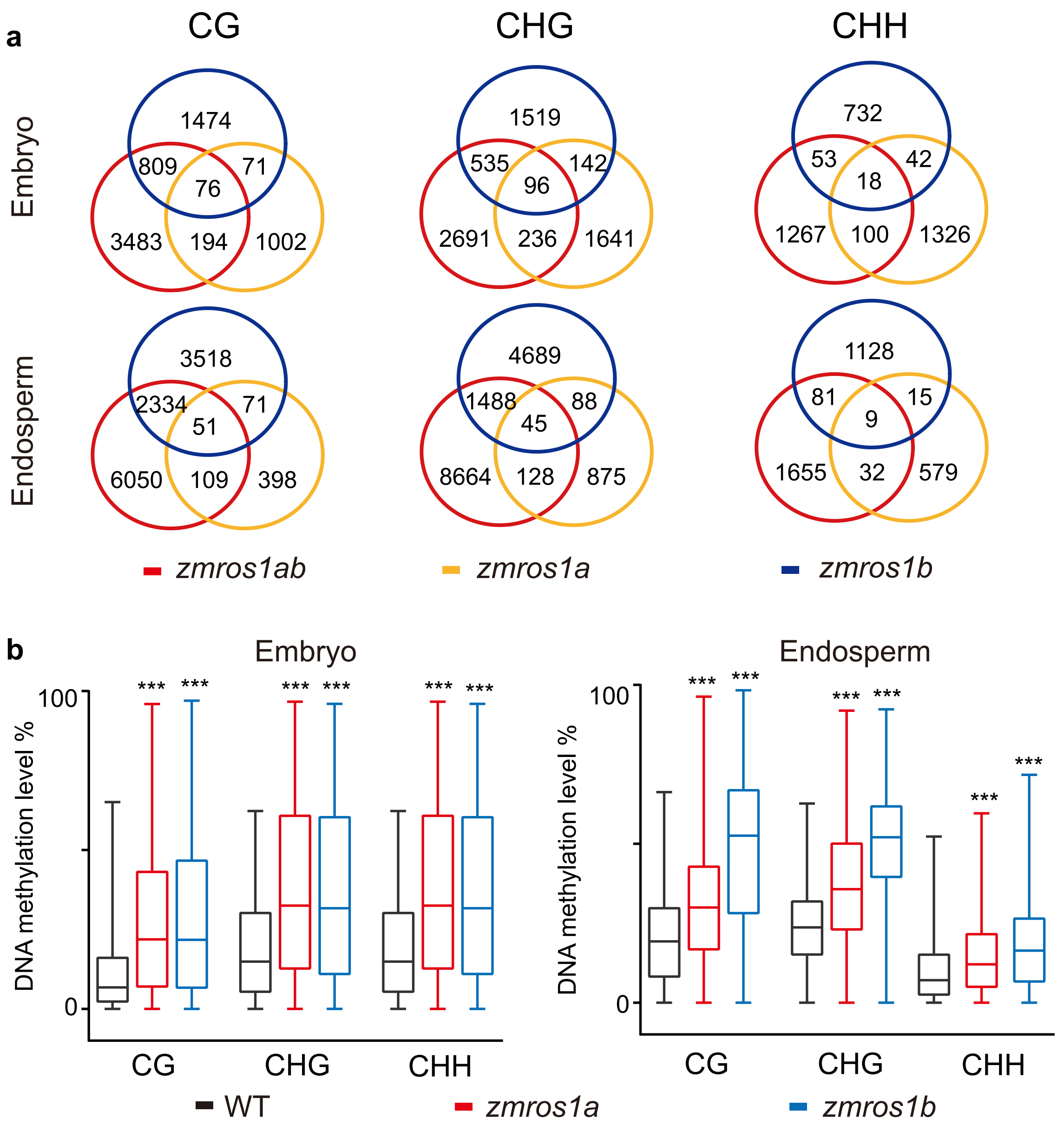

Figure S6 Overlap of DMRs among the single and double mutants. (**a**) Comparison of the DMRs that are identified in the single and double mutants. (**b**) The box plot to show DNA methylation levels in the single mutants of the DMRs that are only identified in *zmros1ab*.

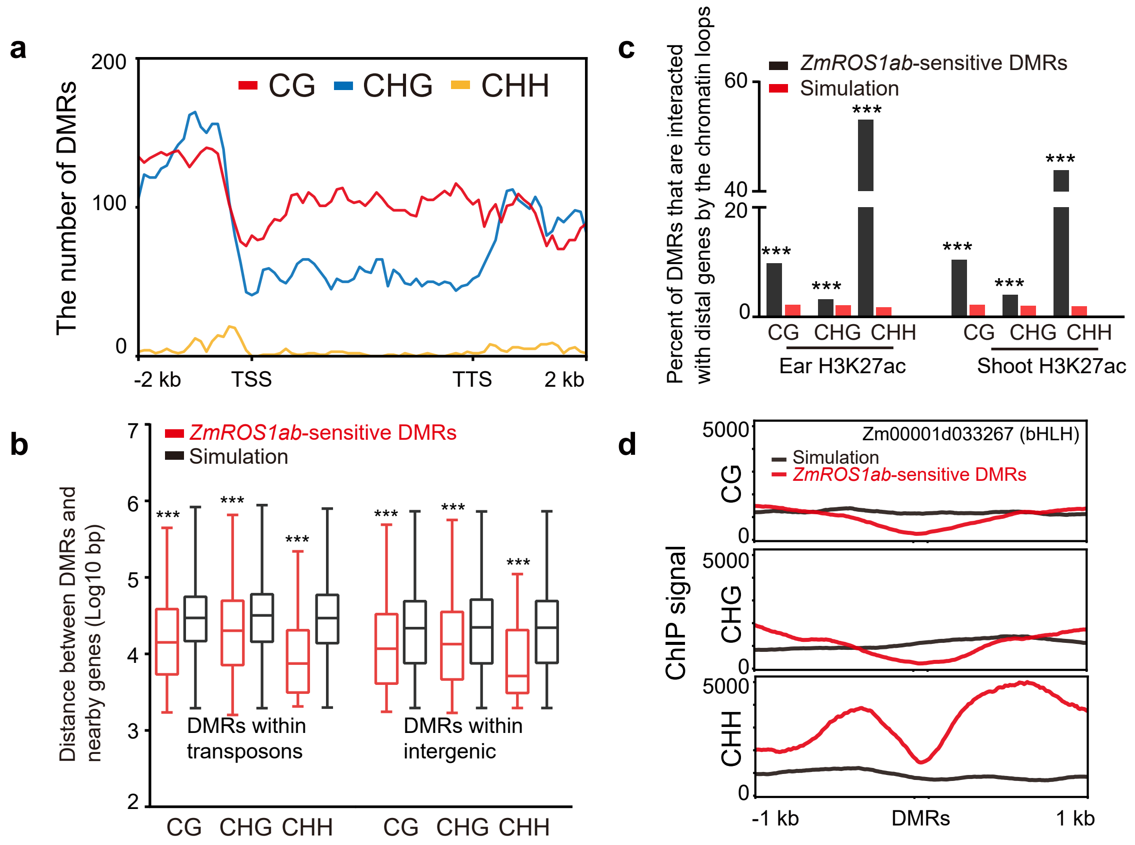

Figure S7 Features of *ZmROS1ab*-sensitive DMRs in endosperm. (**a**) Metaplot to show DMR distribution around genes. (**b**) Boxplot to show distance between DMRs overlapping transposons or intergenic regions and nearby genes. (**c**) Percent of DMRs that are interacted with distal genes by the chromatin loops. ***, P<0.001. (**d**) Enrichment of TF binding around the DMRs with chromatin loops.

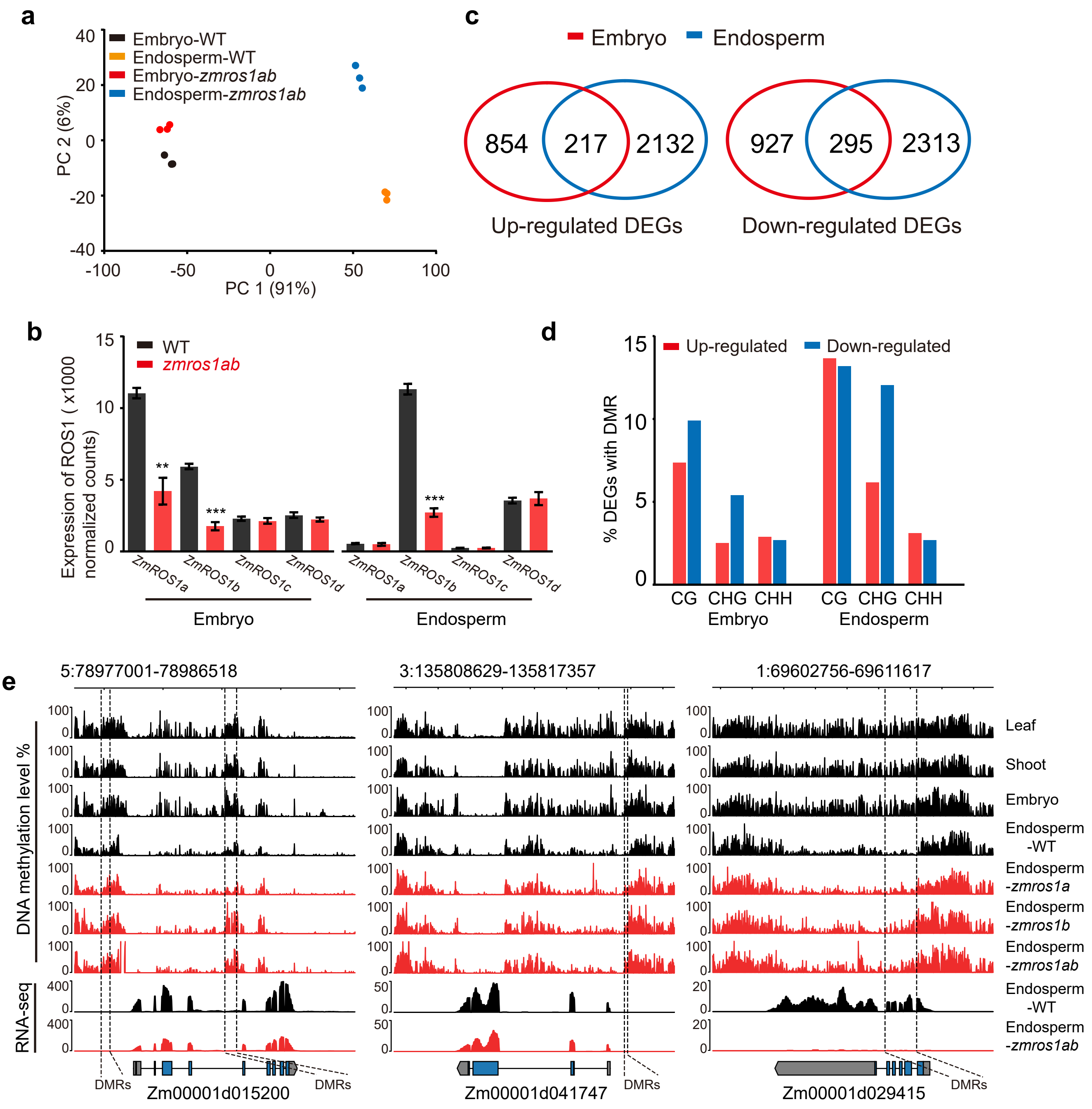

Figure S8 Gene expression analysis in embryo and endosperm. (**a**) PCA analysis of all 12 samples. (**b**) Expression of *ZmROS1a-1d*. (**c**) Overlap of DEGs in embryo and endosperm separated by up- or down- regulation. (**d**) Bar plot to show the percent of DEGs with hypermethylated DMRs in *zmros1ab*. (**e**) Examples to show DNA methylation level and expression level of genes that are preferentially expressed in endosperm. The position of the DMRs is indicated with dashed vertical lines.

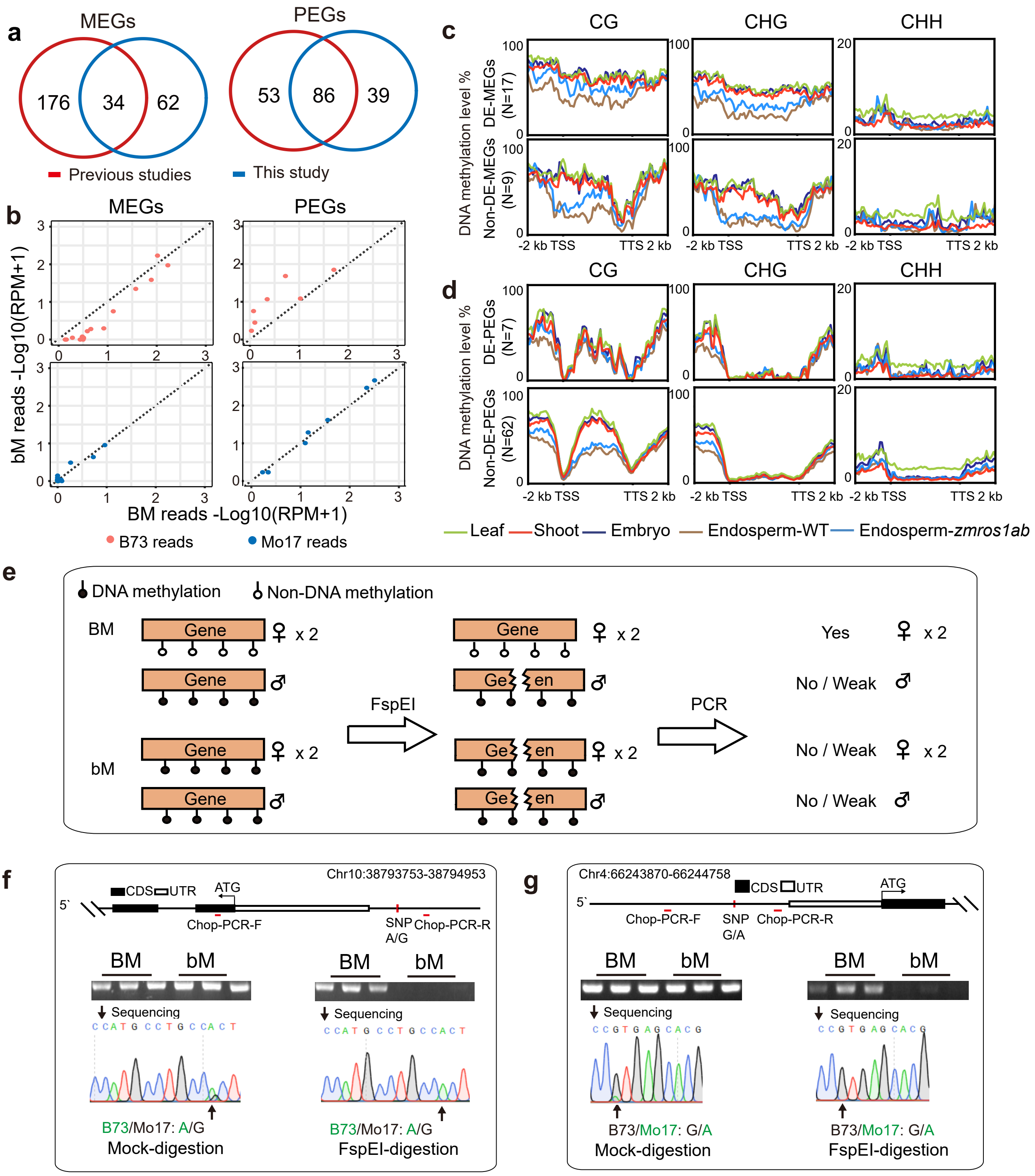

Figure S9 Methylation changes around imprinted genes. (**a**) Comparison of MEGs and PEGs identified in this study and previous studies. (**b**) Comparison of the allelic expression levels in the bM and BM hybrid. RPM, reads per million. (**c-d**) Metaplot to show DNA methylation levels across different tissues for the MEGs (**c**) and PEGs (**d**). DE, differentially expressed between bM and BM. The number of genes used for this analysis was shown in the figure. (**e**) A schematic to show how the two alleles response to FspEI digestion and PCR amplification in BM and bM hybrids. (**f**) Failure to remove DNA methylation from the maternal allele leads to complete DNA cut and no PCR amplification of a MEG. On the left panel, both the B73 and Mo17 alleles can be amplified in both the BM and bM crosses since no enzyme was added (mock digestion). On the right panel, the hypomethylated B73 allele in BM can't be digested by FspEI, resulting in PCR amplification in the BM cross. However, in bM cross, both the B73 and Mo17 alleles are methylated and can be digested, leading to no PCR amplification. (**g**) Failure to remove DNA methylation from the maternal allele leads to complete DNA cut and no PCR amplification of a PEG.

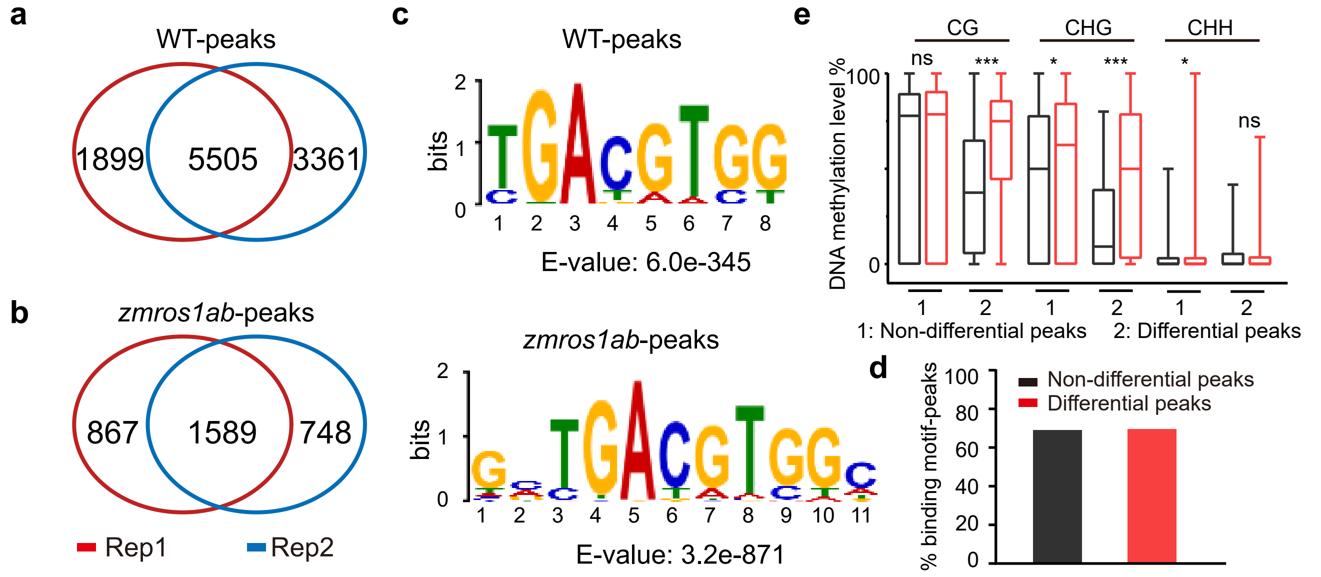

f

	Second experiment		First experiment		Second experiment		First experiment	
Peaks	WT-rep1 37276	WT-rep2 38602	WT-rep1 7489	WT-rep2 8965	*zmros1ab*-rep1 34772	*zmros1ab*-rep2 37571	*zmros1ab*-rep1 2487	*zmros1ab*-rep2 2364
Overlap peaks	30348		5565		29359		1608	
	5390				1602			

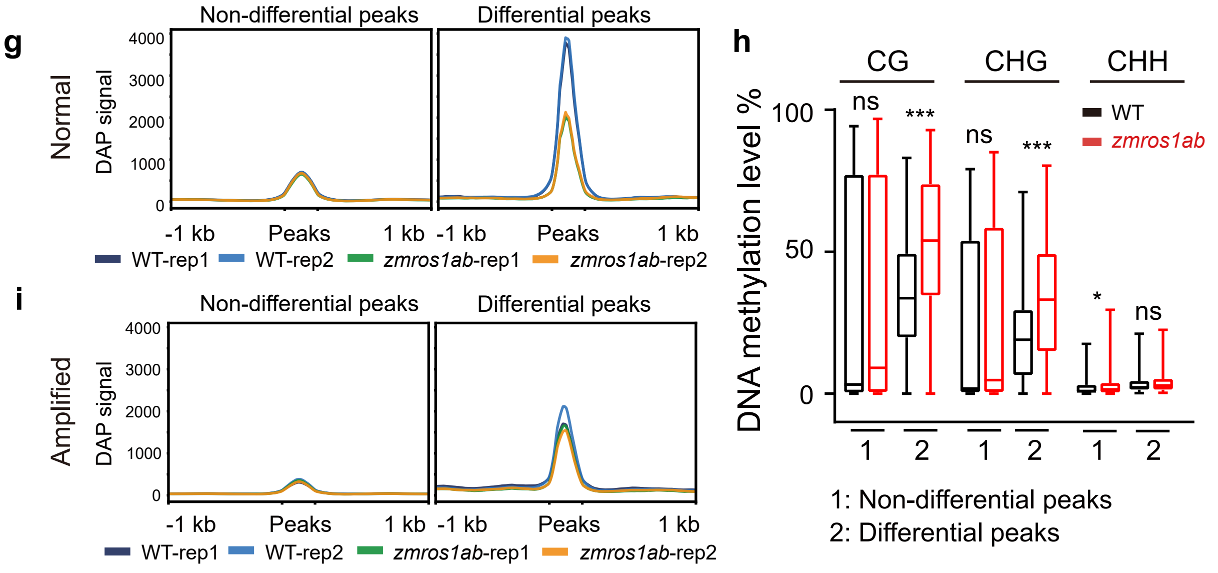

Figure S10 Analysis of ZmO2 peaks. (**a-b**) Comparison of the peaks identified in the two replicates for the WT (**a**) or *zmros1ab* mutant (**b**). (**c**) The binding motifs identified in the peaks in WT and mutant. (**d**) Comparison of the percentage of peaks with the identified binding motif between the differential and non-differential peaks. (**e**) Comparison of methylation levels between the differential and non-differential peaks within the binding motif. (**f**) Overlaps of the ZmO2 binding peaks between two independent experiments. The DAP-seq experiments were performed twice. Results shown in **Figure 6** and **Figure S10** (**a-e**) were from the first experiment. This experiment was replicated using a different biological sample (second experiment). Besides, to remove the methylation difference between the WT and mutant DNA, the PCR amplified sample of both the WT and mutant DNA samples were included for DAP-seq as well. (**g**) Identification of differential peaks between WT and *zmros1ab* mutant in the second experiment. (**h**) Comparison of DNA methylation levels in the differential and non-differential peaks between WT and *zmros1ab* mutant in the second experiment. (**i**) The binding difference of ZmO2 on WT and *zmros1ab* DNA disappear after DNA methylation difference was removed by PCR amplification.

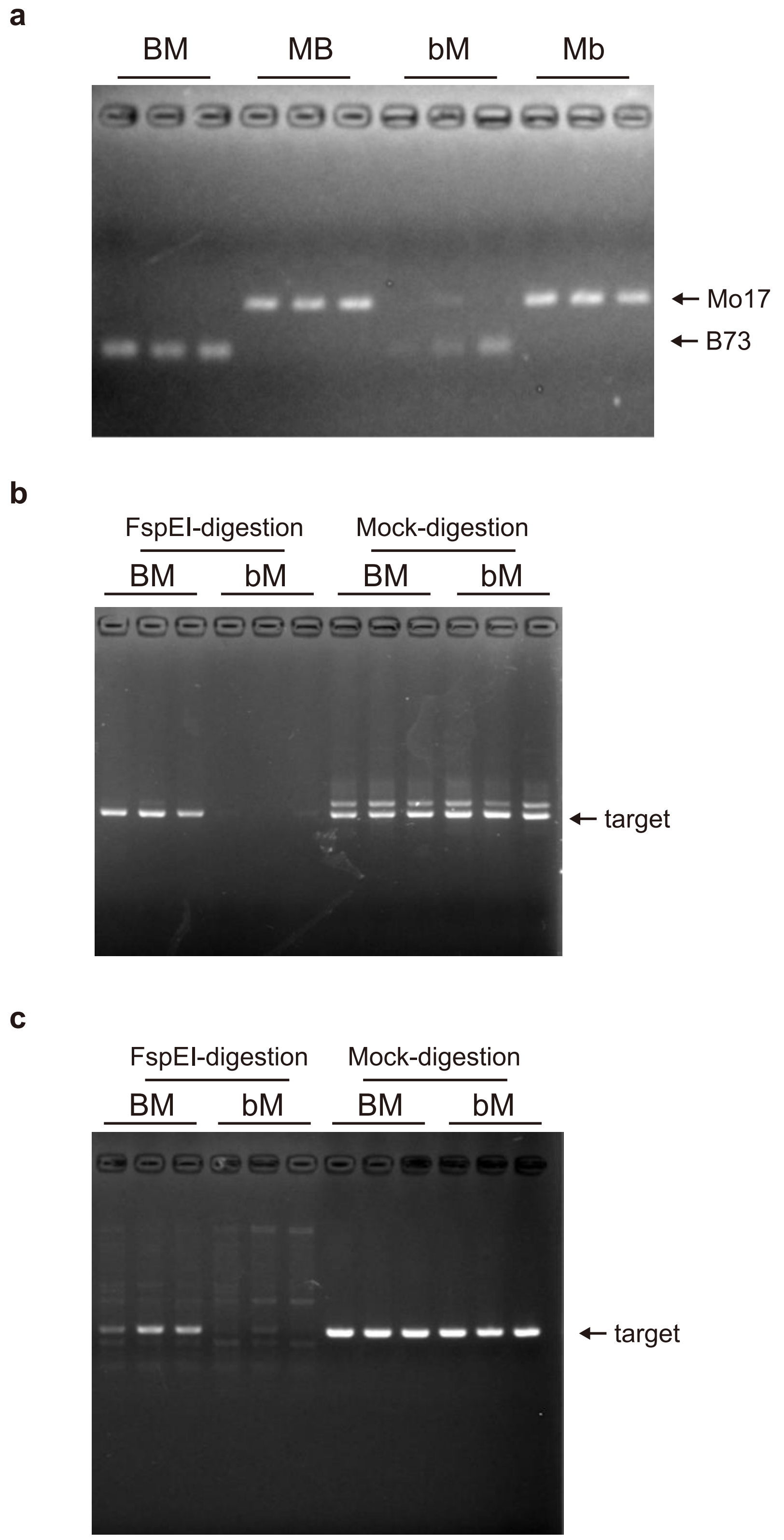

Figure S11 The uncropped agrose gel images that are showed in Fig. 5a (a), Fig. S9f (b) and Fig. S9g (c). The black arrows indicate the position of the target band.

Table S1 Summary of DMRs

Tissue	Genotype	Context	Tiles_total	Tiles_enough_coverage (3X)	DMRs	Hyper_DMRs_number	Hyper_DMR_%
Embryo	*zmros1a*	CG	16,633,602	6,664,705	1,579	1,346	85.2
		CHG	17,550,965	6,812,755	3,139	2,116	67.4
		CHH	19,333,532	12,412,856	2,482	1,486	59.9
	zmros1b	CG	16,657,393	6,865,791	4,329	2,433	56.2
		CHG	17,581,099	7,014,813	4,934	2,289	46.4
		CHH	19,357,225	12,751,616	2,685	845	31.5
	zmros1ab	CG	16,618,532	6,711,878	5,394	4,562	84.6
		CHG	17,534,952	6,861,395	5,518	3,558	64.5
		CHH	19,320,602	12,516,202	2,955	1,438	48.7
Endosperm	*zmros1a*	CG	16,580,869	5,637,871	1,108	630	56.9
		CHG	17,493,846	5,854,631	3,150	1,136	36.1
		CHH	19,288,209	11,502,921	2,335	635	27.2
	zmros1b	CG	16,628,168	5,785,167	7,128	5,974	83.8
		CHG	17,545,239	6,000,996	7,718	6,315	81.8
		CHH	19,331,246	11,765,305	2,415	1,233	51.1
	zmros1ab	CG	16,595,853	5,130,866	9,047	8,544	94.4
		CHG	17,498,717	5,338,391	11,090	10,325	93.1
		CHH	19,304,079	10,734,267	2,762	1,777	64.3

Table S2 Primers used in this study

Type	Primers name	Sequence
EMS	ZmROS1a-emsF	AATGTGATAGTCTCCCCCACA
	ZmROS1a-emsR	ATTGAAGATTTTTATGAAGATGGTG
	ZmROS1b-emsF	GGGTGAGGGATCTAACAACT
	ZmROS1b-emsR	GAATCCATCCTCAGAAAACTAGATT
qRT-PCR	qZmROS1a-F	TGGGAGCATTGACCTTGAGT
	qZmROS1a-R	AGGAAGAGGTTGAATGGGCA
	qZmROS1b-F	ACACGGATTACAGGCAGACT
	qZmROS1b-R	GGGGTGTCGTGTGTGATCTA
	qZmROS1c-F	AACATTTTGGCTGTGCGGAT
	qZmROS1c-R	ACCCATCCTAGCCTTGTCAC
	qZmROS1d-F	TTAGGCAAGCAGAAGTCCGA
	qZmROS1d-R	GAGTCCAAGCCCTCTAATGC
RT-PCR	MEG-RTPCR-F	CTGCTCAAGTCCAACTGCTT
	MEG-RTPCR-R	ACCTCCTTTACCAAACAGAAACA
	PEG-RTPCR-F	GCCAAGCATTCCAAGGTCTG
	PEG-RTPCR-R	TCTGTCTTCGCACCACTGAT
Chop-PCR	MEG-ChopPCR-F	CGATTCCTCCGGTGGTACA
	MEG-ChopPCR-R	CACGTCGCCTTAATTGGGAC
	PEG-ChopPCR-F	AATAATGGCCATGCAGCGC
	PEG-ChopPCR-R	ACGGAATCATATGGACGGCT
BS-PCR	MEG-BS-F	TTAGGCTTGAGGAGTGGYAGGYATGG
	MEG-BS-R	TGACCAATRAARRATCCAAACRCCCC
	PEG-BS-F	TGAACTYGAYAAYAATGGTGGYGTAT
	PEG-BS-R	TACTTCCAATTAATRCTCTCCRTRCTC
