# Supplementary material for: DNA demethylation affects imprinted gene expression in maize endosperm
Source: Genome Biol. 2022 Mar 9;23:77. doi: 10.1186/s13059-022-02641-x (PMC8905802; doi:10.1186/s13059-022-02641-x)
Supplement: Supplementary file 7 — Additional file 7. Review history. [file 13059_2022_2641_MOESM7_ESM.docx]

Review History

**First round of review**

**Reviewer 1**

**Are you able to assess all statistics in the manuscript, including the appropriateness of statistical tests used?**

Yes, and I have assessed the statistics in my report.

**Comments to author:**

Qing Li and colleagues present data here which shows that DML orthologs in maize carry out demethylation in the maternal gametophyte/sporophyte and that this demethylation is required for imprinting. This work is an important contribution as it extends the model derived from Arabidopsis to maize. This was a fun read and should be published. I have one major comment and a few minor comments and some suggestions for experiments or analysis. Implementation of the analyses listed under minor comments will help improve the paper but are not necessary for the publication of the paper.

Major comments.

1. I find Chop-PCR to be unreliable and hard to interpret. The Chop-PCR assay carried out under Figure 5 with FSPE1 and the sanger sequencing traces shown underneath them are nearly meaningless. Bisulfite PCR and sanger sequencing or sanger sequencing after enzymatic treatment using the EM-Seq kit available from NEB are more reliable.

Minor comments.

1. I urge the authors to make the figures more readable to people who have issues with identifying colors (that includes me). Here are a few examples of where they color schemes can be improved. In Fig2A and B, the color of the text for Hyper and hypo are hard to distinguish. Please change colors and add squares to indicate the color.In fig 3F, the colors and density of the plots make the figure nearly uninterpretable. It might even help there to show fewer tisuues.

2. The authors should mention the source of the data for Fig S1C. Also, the axis in S1C probably doesn't need "of".

3. What is the source of the EMS mutants. Where/how were they obtained?

4. What is the conversion rate from the bisulfite treatment?

5. The phenotypic analysis suggests that these mutants have no seed abortion. This is in contrast to the loss of DME in other species. The authors should speculate in discussion on why there is no abortion.

6. On page 12, line 238, PCA is written as principle component analysis. Its principal, right?

7. What is the significance of the chromatin loops? Are these loops enriched in TFBS?

8. Williams et al https://journals.plos.org/plosgenetics/article?id=10.1371/journal.pgen.1005142 have previously suggested that ROS1 expression in maize may also be positively regulated by ROS1 in a rheostat. I am curious if you see evidence for this in your data. Also, do you see a DMR associated with the ROS1 genes?

9. The CHG hypermethylation in intriguing. Are genes involved in laying down CHG methylation upregulated in ROS1ab mutants? Is the CHG hypermethylation associated with sRNAs?

10. On Page 13, lines 252-254, the authors state that " DMRs can be connected with 1983…..". Authors should define what connect mean. Are these DMRs within 1Kb/2Kb/3Kb of a gene?

11. CHG hypermethylation in the endosperm has been previously described by Klosinska et al (2013) https://www.nature.com/articles/nplants2016145 and Moreno-Romero et al (2019) https://link.springer.com/article/10.1186/s13059-019-1652-0 . Please refer to their work.

12. On Page16, line 319, the authors refer to "gene expression is exerted through the maternal allele (i.e. the central cell). If this is the case, that would be the maternal gametophyte. I am curious if the authors have looked at any ovule data(if it should exist) to show that ROS1a/b are expressed in these tissues.

13. If you run your imprinting pipeline on wildtype and maternal mutant data, can you identify genes that were biallelically expressed in wild-type but are mono-allelically expressed in mutants where the ros1 mutant is maternally inherited. This would show the importance of ROS1 at non-imprinted genes in wildtype.

14. In Fig S10E, there is a spelling mistake in Differential on one of the X-axes.

15. Since DAMID is an invitro assay, the authors can test if CG DNA methylation is important for TF binding by methylating the DNA with M.SssI.

**Reviewer 2**

**Are you able to assess all statistics in the manuscript, including the appropriateness of statistical tests used?**

Yes, and I have assessed the statistics in my report.

**Comments to author:**

Although the functions of ROS1 and DEMETER DNA demethylase in Arabidopsis and rice are extensively studied, those in maize are remain unknown. This paper reports the function of maize DNA demethylase using ros1ab double mutants. Maize ros1ab mutants show higher levels of DNA methylation, more down-regulated gene expression compared to WT. ros1ab mutants also showed alteration of imprinted gene expressions that are associated with DMRs and reduced transcription factor binding.

This paper provides some important aspects of the ROS1AB functions. However, I think a few points have to be clarified and adding more data will improve the paper.

My major concern is that

1. Characterization of ros1a and ros1b mutant plants are required if ros1a and ros1b mutant plants are used in this study for the first time;

one example, ROS1a expression in ros1a and ros1b mutant, ROS1B expression in ros1a and ros1b mutants in embryos and endosperms are all prerequisite for using the mutant plants. Are these null alleles or hypo alleles? As in the figure, each gene expression in the double mutants gives some hints about it but if the two genes have genetic interaction, it would show differently in the double mutants. Therefore, general characterization of the mutant alleles are needed before going into the main sections.

They predicted each ROS1A and ROS1B RNA reduction in the double mutants is likely due to nonsense mediated RNA decay, but no data for it to prove. It would be good to show RNA levels in each mutant and better to show the primer position for detection of the RNA in the figure (in addition to RNA-seq).

2. In Arabidopsis and rice, there is a distinctive seed abortion phenotype in the mutants. In the figure, however, it is not clear whether maize also shows that abortion phenotype. More phenotypic analyses should be added to be able to compare with those in Arabidopsis and rice. If there are no abortion in the ros1a and ros1b mutants, it might be better to see whether the other homologs have that mutant phenotype. I think this is evolutionary important.

Although ROS1C and ROS1D are expressed less than ROS1A and ROS1B, we cannot rule out whether they have important functions during embryogenesis.

3. Another concern is for the quality of the methylome data. Based on the following aspects, I suspect whether the data are reliable.

1) In the page 29 method section, the authors removed the low quality of the read as Q<20. But most of the reliable data published use Q<30.

2) Also, they allowed up to five mismatches which is too many. Most of the data use zero or up to one.

3) the genome coverage (3X) of the library is too low.

4. The authors finally pinpoint a few genes or aspects in each result section which is starting from the bulk data (methylation, RNA expression, imprinting and transcription binding). They conclude that is from ROS1AB function. I am not saying this is wrong. But I am saying that there are many more genes that are not fitted in that category. However, the authors always or tend to generalize the conclusion even small number genes or DMRs show while so many others are not. This might mislead the reader's understanding.

Minor concerns are

1. In page 8 line 150, "The mutation abolishes the RPM-fold domain" -> mutation is not RPM domain but in the glycosylase domain.

2. Figure S2, label for a, b, c, d in the legends is missing. Which one is WT and which one are the mutants???

3. Page 9 line 158, ROS1a and ROS1b gene expressions in the embryo and endosperm of each mutant and double mutant compared to WT should be done. Also it might be better to show more detail for the developmental reproduction phenotype (e.g 14 DAP clearing the tissues)

4. Genetic analyses (e.g. transmission of the mutant allele) has to be done more thoroughly when using the new mutants. Rice ros1a mutant allele shows transmission problem and inhertance of the mutant allele cause developmental defects as in the Arabidopsis. Therefore, it is required in this study.

5. In the Figure S1C legends, no label for the stage.

6. In the Figures, red and orange colors are too similar.

7. In the Figure S3 legends, "The 100-bp tiles that have enough coverage were used to perform this analysis." -> 'enough ' is not a precise term.

8. In the page 10 line 180, "in a locus-dependent manner." -> I think it is CHG sites.

9. Page 10 line 211, "39% of CG and 41% of CHG DMRs identified". This is the same when we see in this way " => 61% CG DMR is not hypomethylated in the endosperm compared to embryo. Also 87% (3350/21390) of the hypomethlyated DMRs in the endosperm than embryo are not ros1ab targets.

10. Page 10 line 216 "don't" -> do not

11. Page 13 line 243, I am wondering if the authors did qRT. If so, it is better to show the location of the primer set and the result.

12. Page 13 line 249, there is no legends for the hypermethylated DMR in the Figure S8D.

13. Imprinted gene expression by qRT-PCR in page 18 should be shown (maternal vs. paternal).

14. Page 18 line 362, "The above analyses suggest that ZmROS1ab regulates gene expression and imprinting in maize endosperm." -> again among 96 MEG, a few are in this category.

15. Page 20 line 399, again although there are a few genes show this pattern, it is a little hard to generalize this conclusion to me.

**Authors Response**

**Point-by-point responses to the reviewers’ comments:**

Reviewer #1:

Qing Li and colleagues present data here which shows that DML orthologs in maize carry out demethylation in the maternal gametophyte/sporophyte and that this demethylation is required for imprinting. This work is an important contribution as it extends the model derived from Arabidopsis to maize. This was a fun read and should be published. I have one major comment and a few minor comments and some suggestions for experiments or analysis. Implementation of the analyses listed under minor comments will help improve the paper but are not necessary for the publication of the paper.

*Reply: Thanks very much for the positive comments.*

Major comments.

1. I find Chop-PCR to be unreliable and hard to interpret. The Chop-PCR assay carried out under Figure 5 with FSPE1 and the sanger sequencing traces shown underneath them are nearly meaningless. Bisulfite PCR and sanger sequencing or sanger sequencing after enzymatic treatment using the EM-Seq kit available from NEB are more reliable.

*Reply: This is a useful suggestion; we replaced the Chop-PCR results with the bisulfite PCR and sequencing results in the revised manuscript. The results are similar, namely, the maternal allele is differentially methylated between the wild-type cross (B73 x Mo17) and the mutant cross (b73 x Mo17), while there is no difference in the paternal allele (Figure 5E-5F). The Chop-PCR results were provided in the supplemental figure (Additional file 1: Figure S9C-S9E) as a support of the sequencing results.*

Minor comments.

1. I urge the authors to make the figures more readable to people who have issues with identifying colors (that includes me). Here are a few examples of where they color schemes can be improved. In Fig2A and B, the color of the text for Hyper and hypo are hard to distinguish. Please change colors and add squares to indicate the color. In fig 3F, the colors and density of the plots make the figure nearly uninterpretable. It might even help there to show fewer tissues.

*Reply: We apologize for the color scheme that was not useful for individuals with color issues in the previous versions of the figures. We changed the color schemes of Figures 2A, 2B, and the other figures, simplified Figure 3F by removing three tissues (anther, earshoot and shoot apex) with similar DNA methylation levels. Hopefully these figures will be readily interpretable now.*

2. The authors should mention the source of the data for Fig S1C. Also, the axis in S1C probably doesn't need "of".

*Reply: Thanks for the suggestion. The data for Figure S1C was from the study by Yi et al. (2019). We added this in the figure legend. We also explained in detail the tissues used in this Figure. We removed “of” from y axis label.*

3. What is the source of the EMS mutants. Where/how were they obtained?

*Reply: The EMS mutant was generated by Lu et al. (Gene-indexed mutations in maize. Mol Plant, 2018, 11(3):496-504). These mutants can be obtained through this website, http://elabcaas.cn/memd/. We modified the Methods to make this clearer.*

4. What is the conversion rate from the bisulfite treatment?

*Reply: The conversion rate was between 99.2% and 99.5% for each of the eight libraries used in this study. This was calculated based on the conversion of cytosine sites on the chloroplast genome. This information was included in the Methods section in the revised manuscript and can also be found in Additional file 2.*

5. The phenotypic analysis suggests that these mutants have no seed abortion. This is in contrast to the loss of DME in other species. The authors should speculate in discussion on why there is no abortion.

*Reply: This is a good point and we have expanded the text to speculate on this. The lack of a seed abortion phenotype is most likely due to the redundant functions of the other genes encoding DNA demethylase in maize genome. There are four candidate demethylase encoding genes, named as ZmROS1a, ZmROS1b, ZmROS1c, and ZmROS1d. Here we focused on the two most abundantly expressed genes in embryo and endosperm, ZmROS1a and ZmROS1b. However, it is likely that the other two genes, ZmROS1c and ZmROS1d which are also expressed at lower levels in endosperm, can complement the loss of ZmROS1a and ZmROS1b. We added these speculations to the Discussion.*

6. On page 12, line 238, PCA is written as principle component analysis. Its principal, right?

*Reply: Thanks for pointing this out, we corrected it in the revised manuscript.*

7. What is the significance of the chromatin loops? Are these loops enriched in TFBS?

*Reply: This is significant at a P value of 0.001, these results were included in Figure S7C. To look at whether these DMRs with chromatin loops are enriched for binding sites of any transcription factors, we used public data for the binding sites of 104 transcription factors (Tu et al. Reconstructing the maize leaf regulatory network using ChIP-seq data of 104 transcription factors. Nat Commun, 2020, 11(1):5089). The pattern is quite similar for all 104 TFs. One example is shown below in Figure R1. There is no enrichment of TF binding sites for the CG and CHG DMRs. For CHH DMRs, the binding seems to be higher on the flanking regions of the DMR. It is difficult to explain this phenomenon. Since this pattern was observed for all 104 TFs, it is quite likely that this is not biologically meaningful.*

*Figure R1 Enrichment of TF binding around the DMRs.*

8. Williams et al https://journals.plos.org/plosgenetics/article?id=10.1371/journal.pgen.1005142 have previously suggested that ROS1 expression in maize may also be positively regulated by ROS1 in a rheostat. I am curious if you see evidence for this in your data. Also, do you see a DMR associated with the ROS1 genes?

*Reply: This is an interesting question. This paper suggests an important characteristic of the rheostat, i.e., the expression level of the ROS1 gene was up-regulated in the mutant. Therefore, we investigated the expression levels of ZmROS1 in the mutant to see whether ZmROS1 can regulate itself. No up-regulation was observed in maize (Additional file 1: Figure S2A). However, both zmros1a and zmros1b are nonsense mutations, the nonsense-mediated decay could be a complicating factor in measuring transcript abundance. Also we didn’t see a DMR in the ZmROS1 genes. Therefore, it is unclear whether such a rheostat exists in maize.*

9. The CHG hypermethylation in intriguing. Are genes involved in laying down CHG methylation upregulated in ROS1ab mutants? Is the CHG hypermethylation associated with sRNAs?

*Reply: We would note that the “hypermethylation” here refers to the differences between the WT and the mutant. The CHG “hypermethylated” regions in the mutant compared to wild-type actually are methylated at the same level in the mutant endosperm as they are in other tissues of maize. This largely suggests that what we are seeing is a failure to demethylate rather than an increase in CHG methylation in the mutant. We did also check the expression of the chromomethylase genes, and see an increase in zmros1ab mutant in endosperm, and no major changes in embryo (Additional file 4).*

10. On Page 13, lines 252-254, the authors state that " DMRs can be connected with 1983…..". Authors should define what connect mean. Are these DMRs within 1Kb/2Kb/3Kb of a gene?

*Reply: We apologize for the confusion. Here, the DMRs were connected with genes because they were located within 2kb of a gene, we included this in the revised manuscript.*

11. CHG hypermethylation in the endosperm has been previously described by Klosinska et al (2013) https://www.nature.com/articles/nplants2016145 and Moreno-Romero et al (2019) https://link.springer.com/article/10.1186/s13059-019-1652-0 . Please refer to their work.

*Reply: The reviewer is correct in noting these prior results on CHG hypermethylation in endoserm and we have cited these (Page 12 Line 232). However, we might have not been clear enough in our labels in Figure 2. The vast majority of changes are examples of hypermethylation in embryo compared to endosperm. We only find a small number of regions with higher CHG methylation in the endosperm which is what is referenced in the papers above. We have added details to the figure to make this more clear.*

12. On Page16, line 319, the authors refer to "gene expression is exerted through the maternal allele (i.e. the central cell). If this is the case, that would be the maternal gametophyte. I am curious if the authors have looked at any ovule data(if it should exist) to show that ROS1a/b are expressed in these tissues.

*Reply: This is a good point. We indeed found one manuscript that contains expression data of ovule (Chettoor et al. Discovery of novel transcripts and gametophytic functions via RNA-seq analysis of maize gametophytic transcriptomes. Genome Biol. 2014, 15(7): 414). Both ZmROS1a and ZmROS1b are expressed in the ovule. We added this in the Discussion (Page 25, Lines 505-507).*

*Figure R2 Expression levels of the four ZmROS1 genes in four tissues.*

13. If you run your imprinting pipeline on wildtype and maternal mutant data, can you identify genes that were biallelically expressed in wild-type but are mono-allelically expressed in mutants where the ros1 mutant is maternally inherited. This would show the importance of ROS1 at non-imprinted genes in wildtype.

*Reply: This is a good point. We performed the analysis as suggested. In total, there are 12,524 genes that have enough reads coverage (>= 10 reads for each allele in the wide-type cross) to perform this analysis. After requiring bi-allelic expression in wide-type (The percentage of B73 reads is between 30% and 70%) and mono-allelic expression in mutant (The percentage of the reads from the highest expressed allele is over than 90%), only three genes were found (see following). Therefore, it is likely that ZmROS1ab has a limited role in regulating allele-specific expression of non-imprinted genes.*

*Gene ID	B73xMo17-B73 reads B73xMo17-Mo17 reads	b73xMo17-B73 reads	b73xMo17-Mo17 reads*

*Zm00001d003167	15	31	1	17*

*Zm00001d007026	31	22	2	21*

*Zm00001d008209	29	50	3	38*

14. In Fig S10E, there is a spelling mistake in Differential on one of the X-axes.

*Reply: Thanks for pointing this out. We corrected the mistake.*

15. Since DAMID is an in vitro assay, the authors can test if CG DNA methylation is important for TF binding by methylating the DNA with M.SssI.

*Reply: Thanks for the suggestion. We re-did the experiment using a different biological replicate, and did two technical replicates for each sample. The trends are similar between the two experiments (Additional file 1: Figure S10F-S10H). Besides, we included the PCR amplified sample of both the WT and mutant samples. If the binding difference between WT and zmros1ab mutant was due to differential DNA methylation levels, the amplified samples would show no binding difference as PCR amplification would remove the DNA methylation. Our observation is consistent with the expectation (Additional file 1: Figure S10I).*

Reviewer #2:

Although the functions of ROS1 and DEMETER DNA demethylase in Arabidopsis and rice are extensively studied, those in maize are remain unknown. This paper reports the function of maize DNA demethylase using ros1ab double mutants. Maize ros1ab mutants show higher levels of DNA methylation, more down-regulated gene expression compared to WT. ros1ab mutants also showed alteration of imprinted gene expressions that are associated with DMRs and reduced transcription factor binding.

This paper provides some important aspects of the ROS1AB functions. However, I think a few points have to be clarified and adding more data will improve the paper.

*Reply: Thanks very much for the positive comments.*

My major concern is that

1. Characterization of ros1a and ros1b mutant plants are required if ros1a and ros1b mutant plants are used in this study for the first time; one example, ROS1a expression in ros1a and ros1b mutant, ROS1B expression in ros1a and ros1b mutants in embryos and endosperms are all prerequisite for using the mutant plants. Are these null alleles or hypo alleles? As in the figure, each gene expression in the double mutants gives some hints about it but if the two genes have genetic interaction, it would show differently in the double mutants. Therefore, general characterization of the mutant alleles are needed before going into the main sections.

They predicted each ROS1A and ROS1B RNA reduction in the double mutants is likely due to nonsense mediated RNA decay, but no data for it to prove. It would be good to show RNA levels in each mutant and better to show the primer position for detection of the RNA in the figure (in addition to RNA-seq).

*Reply: This is a helpful suggestion. We analyzed the expression levels of the four ZmROS1 genes in zmros1a and zmros1b single mutants using real-time qRT-PCR (Additional file 1: Figure S2A). The expression of ZmROS1a was significantly reduced in zmros1a single mutant as well as in zmros1ab double mutants in both embryo and endosperm. Its expression in zmros1b single mutant was slightly reduced in embryo but not changed in endosperm. For ZmROS1b, the expression was significantly reduced in both zmros1b single and zmros1ab double mutants in both embryo and endosperm, and it was not changed in zmros1a single mutant. The other two genes, ZmROS1c and ZmROS1d don’t show any expression differences in neither the single nor the double mutants. This was included in the main text (Page 9 Lines 154-162) and was included in Additional file 1: Figure S2A. The regions used for real-time qRT-PCR analysis were labeled using red lines in Additional file 1: Figure S1A.*

*Besides the expression levels, we also investigated the transmission of the single mutant allele (Additional file 1: Figure S2H-S2J). In both the zmros1a and zmros1b single mutants, the homozygous mutant can be recovered in the expected ratio of 1/4 from the selfed heterozygous +/- plants. This suggests that the mutated allele of both ZmROS1a and ZmROS1b can be normally transmitted through egg cells and pollens. Similarly, the double mutant gamete can be normally transmitted through both parents (Additional file 1: Figure S2J).*

2. In Arabidopsis and rice, there is a distinctive seed abortion phenotype in the mutants. In the figure, however, it is not clear whether maize also shows that abortion phenotype. More phenotypic analyses should be added to be able to compare with those in Arabidopsis and rice. If there are no abortion in the ros1a and ros1b mutants, it might be better to see whether the other homologs have that mutant phenotype. I think this is evolutionary important.

Although ROS1C and ROS1D are expressed less than ROS1A and ROS1B, we cannot rule out whether they have important functions during embryogenesis.

*Reply: We completely agree and would have preferred to assess the phenotypes in all four genes but we only had access to the zmros1a and zmros1b mutants initially. We checked whether the mutant allele was transmitted in the expected ratio in the self pollinated ear of the zmros1a/+;zmros1b/+ plants. As shown for major concern 1, the double mutant allele of zmros1a and zmros1b can be transmitted through both parents. However, this doesn’t necessarily mean that these two genes are not important. We noted that the self pollinated ears from the homozygous double mutants of zmros1ab in general have smaller ears with poor seed-setting (Additional file 2: Figure S2F). And the hundred kernel weight of the double mutants is smaller compared to WT (Additional file 2: Figure S2G). This suggests that the two genes are important for seed development. The lack of seed abortion is probably due to the redundant function of the other two genes, ZmROS1c and ZmROS1d, as suggested by the reviewer. Further characterization of mutant alleles of these two genes would help to address this question, but this would take years of work and would complicate the current work. However, as this is an interesting and important question, we will continue to work on this in the future.*

3. Another concern is for the quality of the methylome data. Based on the following aspects, I suspect whether the data are reliable.

1) In the page 29 method section, the authors removed the low quality of the read as Q<20. But most of the reliable data published use Q<30.

2) Also, they allowed up to five mismatches which is too many. Most of the data use zero or up to one.

3) the genome coverage (3X) of the library is too low.

*Reply: We apologize for the misunderstanding brought by these parameters that we used. These parameters were chosen because they were used before (Li et al., PNAS, 2015, 112(47):14728-14733), not because of low-quality of our dataset. Nevertheless, we re-analyzed the data based on the suggested parameters (i.e. Q<30, mismatches ≤1 and coverage ≥ 4). The results are quite similar. As an example, we showed Figure 1C with the new parameters here (Figure R3).*

*Figure R3 DNA methylation levels around genes (upper panel) and TEs (down panel) for CG, CHG and CHH. The suggested parameters were used in this analysis.*

4. The authors finally pinpoint a few genes or aspects in each result section which is starting from the bulk data (methylation, RNA expression, imprinting and transcription binding). They conclude that is from ROS1AB function. I am not saying this is wrong. But I am saying that there are many more genes that are not fitted in that category. However, the authors always or tend to generalize the conclusion even small number genes or DMRs show while so many others are not. This might mislead the reader's understanding.

*Reply: We apologize for the confusions. We added in the end of each section some words to remind the reader that these results may not be generalized to each gene. You can find these sentences in Lines 261-262, 313, 359, 394-395, 400 and 447.*

Minor concerns are

1. In page 8 line 150, "The mutation abolishes the RRM-fold domain" -> mutation is not RRM domain but in the glycosylase domain.

*Reply: We changed the sentence into “The truncated proteins lack the RRM-fold domain in ZmROS1a and both the DNA glycosylase and RRM-fold domains in ZmROS1b”.*

2. Figure S2, label for a, b, c, d in the legends is missing. Which one is WT and which one are the mutants???

*Reply: We apologize for the confusion, a, b, c and d represents the WT, zmros1a single mutant, zmros1b single mutant and the double mutants, respectively. We modified the labels in the figures. Hopefully it looks clearer this time.*

3. Page 9 line 158, ROS1a and ROS1b gene expressions in the embryo and endosperm of each mutant and double mutant compared to WT should be done. Also it might be better to show more detail for the developmental reproduction phenotype (e.g 14 DAP clearing the tissues)

*Reply: Thanks for the suggestion. Gene expression levels based on real time qRT-PCR were added (Additional file 1: Figure S2A). Please refer to major concern 2 for the phenotypes of the mutant kernels. We totally agree that it is a good idea to look at the kernels during developmental reproduction. Unfortunately, we haven’t done this in the past, and will continue to look at this in the future.*

4. Genetic analyses (e.g. transmission of the mutant allele) has to be done more thoroughly when using the new mutants. Rice ros1a mutant allele shows transmission problem and inhertance of the mutant allele cause developmental defects as in the Arabidopsis. Therefore, it is required in this study.

*Reply: We appreciate this suggestion and agree that this is important for this study. We characterized the transmission of zmros1a and zmros1b in single and double mutants. Please refer to reply to major concerns 1 and 2.*

5. In the Figure S1C legends, no label for the stage.

*Reply: Thanks for pointing this out. We added more details about the tissues and developmental stages in the figure legend in the revised manuscript.*

6. In the Figures, red and orange colors are too similar.

*Reply: We apologize for the inconveniences; we changed the color schemes of the figures.*

7. In the Figure S3 legends, "The 100-bp tiles that have enough coverage were used to perform this analysis." -> 'enough ' is not a precise term.

*Reply: We changed the sentence and now it reads “The 100-bp tiles that have enough coverage (3X) were used to perform this analysis.”.*

8. In the page 10 line 180, "in a locus-dependent manner." -> I think it is CHG sites.

*Reply: The reviewer is right that ZmROS1ab has a more profound effect on CHG sites than CG or CHH. Here, we want to say that some genomic regions are more likely to be targeted by ZmROS1ab (i.e. the target regions are not uniformly distributed across the genome). We modified the original sentence to “these results suggest that maize ZmROS1ab doesn’t target the genome equally and may play a role in the reduced methylation levels observed in endosperm relative to other tissues in a locus-dependent manner.”. Hopefully it is clearer this time.*

9. Page 10 line 211, "39% of CG and 41% of CHG DMRs identified". This is the same when we see in this way " => 61% CG DMR is not hypomethylated in the endosperm compared to embryo. Also 87% (3350/21390) of the hypomethlyated DMRs in the endosperm than embryo are not ros1ab targets.

*Reply: This is a complex issue and we apologize for the mis-understanding. If we focus on the CG targets the reviewer is correct that 39% are significant DMRs in both contrasts. However, this does not necessarily mean that the other 61% are not differentially methylated in both contrasts. In some cases we were missing data for one of the two contrasts due to insufficient coverage in one sample. In many more cases there is a difference in methylation levels in both contrasts but the difference did not meet our stringent criteria to be classified as a DMR. This is why we presented the visualization of methylation levels in all four samples in Figure 2D. It is clear that many of the 5189 DMRs that are found only in WT vs zmros1ab or the 18630 DMRs in embryo vs endosperm actually have shared methylation differences in both contrasts but did not reach the significance threshold. To make this more clearer, we added the following sentence in Lines 242-245 “For example, among the 5189 CG DMRs that are only identified in the WT versus zmros1ab comparison, about 1/3 of them show clear hypomethylation in endosperm compared to embryo though not reaching the stringent criteria to be defined as DMRs (Figure 2D).”.*

10. Page 10 line 216 "don't" -> do not

*Reply: We made the changes as suggested.*

11. Page 13 line 243, I am wondering if the authors did qRT. If so, it is better to show the location of the primer set and the result.

*Reply: We added the real time qRT-PCR results in the revised version. Primer position can be found in Additional file 1: Figure S1A and qRT-PCR is in Figure S2A.*

12. Page 13 line 249, there is no legends for the hypermethylated DMR in the Figure S8D.

*Reply: Thanks for pointing this out, we modified the legend and now it reads “Bar plot to show the percent of DEGs with hypermethylated DMRs in zmros1ab.”.*

13. Imprinted gene expression by qRT-PCR in page 18 should be shown (maternal vs. paternal).

*Reply: This is a nice suggestion. We added the expression levels from both RNA-seq and RT-PCR in Figure 5C-5D.*

14. Page 18 line 362, "The above analyses suggest that ZmROS1ab regulates gene expression and imprinting in maize endosperm." -> again among 96 MEG, a few are in this category.

*Reply: Thanks for pointing this out. We changed this sentence to “The above analyses suggest that ZmROS1ab regulates gene expression and imprinting of some genes in maize endosperm.” to remind the readers that not all MEGs are in this category.*

15. Page 20 line 399, again although there are a few genes show this pattern, it is a little hard to generalize this conclusion to me.

*Reply: Thanks for pointing this out. We added “some genes” to remind the readers that these conclusions may not be generalized to all genes.*

**Second round of review**

**Reviewer 1**

The authors have addressed the concerns I have raised and the paper should be published. I have one further comments but the paper need not be returned for further review. The authors must add the conclusions from comments 7 and 8 to the text of the manuscript. I think they are useful points.

Also, they should change line 262 (Page 13) from "that are important for gene regulation" to "that maybe important for gene regulation".

**Reviewer 3**

The manuscript by Wu et al “DNA demethylation affects transcription factor binding and imprinted gene expression in maize endosperm” describes characterization of two maize DNA glycosylase mutants, including phenotype, mutant allele transmission, embryo and endosperm methylome analysis, transcriptome and gene imprinting analysis, DAP-seq analysis, etc. The data presented for the most part seems convincing (except for the nature of the mutants, see below) and the conclusions are largely supported by the results presented. I read it with great interests, along with many questions that need to be clarified. I believe such study will be of interest to the general audience of plant biology and epigenetic fields. However, the current manuscript needs more information in many places to make it easier for the readers to assess and to follow. I few major concerns plus a long list of minor issues should be addressed by the authors.

Major issues:

1. The genesis and propagation of mutant lines for the study was not properly described. Please see below in specific comment for this. In addition, the homozygous mutant generation was not indicated. Were they first generation homozygous plants used in this study? These information are crucial for proper assessment of this manuscript. The website presented where the genetic materials were available was not accessible for some reason. I tried gene name search but was unable to go anywhere. This makes it even more important for the authors to detail these two mutants.

The genetic study presented need more details. I think it is lacking the description of how allele transmission study was conducted. How were the alleles genotyped? I assume this was done in seedling? If seed set is reduced in zmros1ab, how can the allele transmission was as expected?

2. Gent et al recently showed (2021 BioRxiv (https://www.biorxiv.org/content/10.1101/2021.10.04.463062v2) MDR1 (which I believe is ZmROS1b) and dng702 (ZmROS1d) function redundantly in endosperm. Considering ZmROS1a is almost not expressed in the endosperm but both ZmROS1a and ZmROS1b are highly expressed in the embryo, the use of zmros1ab might inadvertently affects methylation levels of sporophyte **tissues where** gametophytes derived from, affecting zmros1ab endosperm methylome. I understand this is an intrinsic issue of the homozygous mutants. The authors can do a better job detailing the mutants and discussing potential pitfalls of the data.

The contribution of ZmROS1a to endosperm methylation seems minimal compared with ZmROS1b (Table S1), yet Fig S4 mCG endosperm gene metaplot both mutants have very similar profiles but with reduced methylation compared with wildtype endosperm. This is apparently different from DMR data presented. This requires explanation and should be analyzed more carefully. Perhaps a comparison between zmros1ab and zmros1b DMRs might be informative for this purpose?

3. The DAP-seq results are in vitro in nature. Although attempts were made to link to in vivo functions by assessing relationship between binding peaks and DEGs, they are nonetheless association in nature. I would suggest toning down the title regarding DNA demethylation affects TF binding part.

Specific comments:

Lines 79-80: I believe Schoft et al PNAS 2011 was the first to report DME’s expression in the vegetative cell of pollen.

Lines 111-113: suggest adding “in the next generation” at the end of the sentence to make this statement clearer.

Fig S1A: Maize gene ID should be labeled here for easy identification.

Fig S1B: how was the tree constructed? It was briefly mentioned in the figure legend but not described in the Methods section. Was the full-length protein sequence used?

Line 150-: how were the EMS mutant lines identified and propagated was not described in sufficient details in the Methods. The Lu et al 2018 I believe was a heavily mutagenized population where exon capture sequencing was used to generate gene-indexed mutations. This is similar to the tiling approach used in Arabidopsis and other organisms. The identified mutant plants should be backcrossed multiple generations to cleanup other unwanted mutations. The authors should describe how this part was done and what measurements were taken to ensure no addition mutations exist in each mutant line used in this study.

Fig 1, CDS as black box. But the NTD conserve domains are also indicated as black boxes. This should be changed.

Fig S1C: the x-axis labels were missing, but with descriptions in legends such asu embryos (10-38 DAP) or Other tissues. I would suggest to re-make this into multiple panels with better labeling of sample stages and tissue source. This is an important information on how the 4 genes are expressed among different stage or tissue. For example, BS-seq samples were done using 14 DAP embryo and endosperm because of high expression of both genes at this stage (lines 177-180).

Which generation of homozygous mutants were used for methylome study?

Fig S2A: I am curious why ZmROS1b expression is reduced in zmros1a mutant embryo? Also what test was done here should be given.

Lines 168-175: I am puzzled if zmros1ab has poorer seed-setting rate, why the allele transmission rate is as expected? I assume the p value was from chi square test, right? Also the genotyping was done in kernels? Or in seedlings? Fig S2C-G, is it possible to assess seed set rates for each mutant? I believe this can be informative. The point is to clarify whether double mutant has gametophytic or early zygotic defects that were missed.

Fig 1C: the CHH panels y-scale should be changed to reveal CHH details. It is impossible to see any minor differences in current figure. This applies to Fig S4 as well. CHH methylation is an important readout for RdDM activity and could inform DME-like activity in the maize embryo and endosperm.

Fig S4: for embryo CG gene metaplot, why the zmros1b causes reduced in coding sequence CG methylation whereas zmros1a (which expressed at higher level than ZmROS1b in embryo) has almost no effect? In endosperm CG metaplot, why both single mutants cause CG methylation reduction whereas double mutant has elevated methylation?

Lines 195-197 and Fig S5: I have a very hard time seeing any meaningful differences between these TEs. The authors should at least point out which ones they were referring to. Perhaps blowup relevant ones in Fig S5 to draw readers’ attention to make their point.

Lines 210-213: I wonder why this is the case for endosperm methylome if ZmROS1a is not/lowly expressed in the endosperm and zmros1a does not yield too many DMRs. My point is that the statement here seems overly generalized. There could be alternative explanation other than redundancy, at least in the endosperm that worth looking into. For example, Fig S6A endosperm CG DMRs, there are 6000 amros1a-specific DMRs, what’s their methylation levels in wt and in single mutants?

Lines 219-221: I am having a hard time understanding what the message the authors were trying to convey here. Fig S6C shows there are partial overlaps between CG and CHG DMRs. What does this even mean biologically? This kind of presentation is descriptive and relatively meaningless without providing rational of the comparison.

Lines 236-238: what’s the point of this convoluted and very confusing statement? They are shared DMRs, why do they have to exhibit a reduced methylation relative to embryo??

Lines 242-245: I believe a reasonable interpretation of the result is that another DNA glycosylase (most likely ZmROS1d, per Gent et al 2021 BioXriv) functions in maize central cell/endosperm.

Fig S7B: what’s the y-axis unit for log10 transformation?

Fig S8B: what is the y-axis unit?

Fig 3C: x-axis label (tissues and stages) should be given.

Fig 3D-E: this is an interesting way of assessing endosperm-preferred or -specific expression. However, current description of the analysis provided in the Methods was rough and the figure label is confusing. The x-axis has an increasing label of endosperm-specific expression, ranging from 0.5 to 0.9? What is the y-axis %? I am trying to guess each column represent a list of genes with certain % of endosperm specificity? And among them, some % of them are associated with DMRs? I think this needs to be better presented. The # of genes in each category should be given. Also, the analysis results (all genes with expression score or ratio) should be included as a supplemental file.

Shouldn’t a parallel set analysis be done for embryo genes as well as a control?

Fig 3F: plots should be enlarged.

Fig 3G: this looks very nice. I would very much like to see single mutant methylation tracks added. And if possible, should a few more examples in a supplemental figure.

Line 315: should specify in maize endosperm. If in Arabidopsis, a whole different set of references should be included here.

Fig 5A and B: the main text is a bit confusing and seems different from Fig legend. Endo-MEGs are the 17 MEGs differentially expressed (line 341)? Perhaps giving gene numbers (N=xx) will help with clarification. Perhaps Fig 5A&B should be moved to supplementals?

Fig S9C&D: it will be nice to indicate where is the SNPs and Chop-PCR primers are located (promoter, coding, etc).

Line 451-452: maize should be included in the sentence here. If also include Arabidopsis, a whole new set of references should be added.

Line 461: cite the Fig for easy reference.

Line 468: change specifically expressed to preferentially expressed for better accuracy.

Line 470: pretty sure Park 2017 isn’t the right reference for this statement. I think Ibarra et al 2012, Slotkin et al 2009, and Kinoshita et al 2004 should be included as well.

Line 476: is Opague2 a known imprinted gene? If so, cite reference. I think the choice of Opague2 for DAP-seq can be better rationalized in the DAP-seq result section.

Lines 505-507: ZmROS1b is highly expressed in endosperm. I think the discussion here should not ignore this fact.

Line 509: Part et al 2017 is not the right reference for here. Did you mean Park 2016? I think Hsieh et al 2011 PNAS is a more appropriate reference for this.

Lines 505-506: PMID: 34185870, 26400170, 30589221 can be added to strengthen your reasoning.

Lines 560-: Suggest changing to “The methylation changes in zmros1ab mutant can affect transcription factor binding…..”.

**Authors Response**

**Point-by-point responses to the reviewers’ comments:**

Reviewer #1:

The authors have addressed the concerns I have raised and the paper should be published. I have one further comments but the paper need not be returned for further review. The authors must add the conclusions from comments 7 and 8 to the text of the manuscript. I think they are useful points.

Reply: Thanks for the nice suggestion; we have added these results into the manuscript (Additional file 1: Figure S7D; Lines 266-272; Lines 286-289).

Also, they should change line 262 (Page 13) from "that are important for gene regulation" to "that may be important for gene regulation".

*Reply: Thanks for the nice suggestion; we have revised the texts as suggested (Line 274 in the revised manuscript).*

Reviewer #3:

The manuscript by Wu et al “DNA demethylation affects transcription factor binding and imprinted gene expression in maize endosperm” describes characterization of two maize DNA glycosylase mutants, including phenotype, mutant allele transmission, embryo and endosperm methylome analysis, transcriptome and gene imprinting analysis, DAP-seq analysis, etc. The data presented for the most part seems convincing (except for the nature of the mutants, see below) and the conclusions are largely supported by the results presented. I read it with great interests, along with many questions that need to be clarified. I believe such study will be of interest to the general audience of plant biology and epigenetic fields. However, the current manuscript needs more information in many places to make it easier for the readers to assess and to follow. I few major concerns plus a long list of minor issues should be addressed by the authors.

*Reply: Thanks very much for your interest.*

Major issues:

1. The genesis and propagation of mutant lines for the study was not properly described. Please see below in specific comment for this. In addition, the homozygous mutant generation was not indicated. Were they first generation homozygous plants used in this study? These information are crucial for proper assessment of this manuscript. The website presented where the genetic materials were available was not accessible for some reason. I tried gene name search but was unable to go anywhere. This makes it even more important for the authors to detail these two mutants.

The genetic study presented need more details. I think it is lacking the description of how allele transmission study was conducted. How were the alleles genotyped? I assume this was done in seedling? If seed set is reduced in zmros1ab, how can the allele transmission was as expected?

*Reply: These are good questions and we apologize for not describing it more clearly. These mutants were obtained from Lu et al. (2018, Mol Plant, 11:496-504) via the website (http://elabcaas.cn/memd/). We are not sure why it is not accessible, please try again, there may be occasional shutdown problems (It was accessible on Dec 8, 2021 as we tried). These mutants were generated using EMS mutagenesis in B73 background as part of a large-scale generation of a sequence indexed resource. The details of the generations for each sample have been added to the methods section. Each of the mutant stocks was maintained in the homozygous mutant background for at least two generations prior to sampling. The genotyping for these alleles is performed using CAPS markers (details provided in Additional file 1: Table S2).*

*The reviewer asks a good question regarding the observation of reduced seed set for the double mutant while also observing no evidence for biased transmission. We do not see evidence for bias in the transmission of either mutant allele. The reduced seed set is likely due to reduced vigor of the double mutant plants. These plants are also shorter than single mutants or wild-type. The reduced vigor leads to overall reduced seed numbers but not necessarily due to lack of transmission of any specific genotype.*

*These information has been added in the revised manuscript (Lines 171-174; Lines 614-641).*

2. Gent et al recently showed (2021 BioRxiv (https://www.biorxiv.org/content/10.1101/2021.10.04.463062v2) MDR1 (which I believe is ZmROS1b) and dng702 (ZmROS1d) function redundantly in endosperm. Considering ZmROS1a is almost not expressed in the endosperm but both ZmROS1a and ZmROS1b are highly expressed in the embryo, the use of zmros1ab might inadvertently affects methylation levels of sporophyte tissues where gametophytes derived from, affecting zmros1ab endosperm methylome. I understand this is an intrinsic issue of the homozygous mutants. The authors can do a better job detailing the mutants and discussing potential pitfalls of the data.

*Reply: Thanks for the suggestion. We added details of the mutants (see major concern 1 and Lines 614-641 in the revised manuscript), and discusses potential pitfalls of the data in the Discussion section (Lines 479-482).*

The contribution of ZmROS1a to endosperm methylation seems minimal compared with ZmROS1b (Table S1), yet Fig S4 mCG endosperm gene metaplot both mutants have very similar profiles but with reduced methylation compared with wildtype endosperm. This is apparently different from DMR data presented. This requires explanation and should be analyzed more carefully. Perhaps a comparison between zmros1ab and zmros1b DMRs might be informative for this purpose?

*Reply: Thanks for pointing this out. We checked our analysis carefully and found that this was caused by inclusion of different 100-bp tiles in making the metaplot. When we were calling DMRs and plotting methylation levels, we first identified 100-bp tiles with enough reads coverage (at least 3X) and at least 6 cytosine sites. In the prior figure we had performed this filtering separately for each sample, resulting in different subsets of data for each genotype. We revised this plot to utilize only 100bp tiles with data available for all samples in the current version. In the revised version there is a difference in mCG levels for zmros1b but not zmros1a compared to the wild-type sample.*

3. The DAP-seq results are in vitro in nature. Although attempts were made to link to in vivo functions by assessing relationship between binding peaks and DEGs, they are nonetheless association in nature. I would suggest toning down the title regarding DNA demethylation affects TF binding part.

*Reply: Thanks for the nice suggestion. We revised the title and the contents related to DAP-seq (Title; Line 44; Lines 415-416; Line 462; Line 543; Lines 560-562; Line 568).*

Specific comments:

Lines 79-80: I believe Schoft et al PNAS 2011 was the first to report DME’s expression in the vegetative cell of pollen.

*Reply: Thanks for pointing this out; we added this reference (Line 80).*

Lines 111-113: suggest adding “in the next generation” at the end of the sentence to make this statement clearer.

*Reply: Thanks for the suggestion; we revised as suggested (Line 113).*

Fig S1A: Maize gene ID should be labeled here for easy identification.

*Reply: Thanks for the suggestion; we labeled the genes as suggested.*

Fig S1B: how was the tree constructed? It was briefly mentioned in the figure legend but not described in the Methods section. Was the full-length protein sequence used?

*Reply: We apologize for the unclarity. We used full-length protein sequence to construct this tree. We added more details in the Materials and Methods section (Lines 597-600).*

Line 150-: how were the EMS mutant lines identified and propagated was not described in sufficient details in the Methods. The Lu et al 2018 I believe was a heavily mutagenized population where exon capture sequencing was used to generate gene-indexed mutations. This is similar to the tiling approach used in Arabidopsis and other organisms. The identified mutant plants should be backcrossed multiple generations to cleanup other unwanted mutations. The authors should describe how this part was done and what measurements were taken to ensure no addition mutations exist in each mutant line used in this study.

*Reply: Please see major concern 1 for the details of the propagation of the materials used in this study. The reviewer is right that the EMS mutant may contain many background mutations. To assess how these might affect the analysis presented, we identified SNPs using our RNA-seq datasets. The three biological replicates of each sample were pooled together to call SNPs using GATK HaplotypeCaller of Genome Analysis Toolkit (GATK, v3.7) with “--filter_reads_with_N_cigar” (McKenna et al., 2010). Next, SNPs were filtered out using the GATK VariantFiltration with the following parameters “QUAL < 30.0; QD < 2.0; MQ < 40.0; FS > 40.0; haplotype score > 13.0; MQRankSum < –12.5; ReadPosRankSum < –4.0”. The remaining SNPs covered by more than thirty reads were kept as confident SNPs. In total, we identified 576 non-redundant homozygous SNPs in the four samples (embryo and endosperm of WT and zmros1ab mutant) located within 364 genes. Among these genes, six has a putative annotation related to epigenetic regulation. Two of the genes are ZmROS1a and ZmROS1b. The other four genes are Zm00001d035228, Zm00001d016340, Zm00001d021910, and Zm00001d011490. The SNP within Zm00001d035228 is the same between the WT and the zmros1ab mutant, while the SNPs within the other three are polymorphic between WT and the zmros1ab mutant. The Zm00001d016340 gene has a SNP that is not within the coding region. The Zm00001d021910 has five SNPs with one being a non-synonymous mutation (Thr to Ala), but this gene is undetectable in seed, endosperm and embryo (FPKM < 0.1). The Zm00001d011490 gene also has a non-synonymous mutation (Glu to Lys). This mutation is not within protein domains. And the close homolog of this gene in maize genome (Zm00001d008941) has a corresponding Ser at the mutated position. Also this amino acid is not conserved in Arabidopsis and rice. Based on all of this, we concluded that there are many background mutations in the mutant but these mutations are not likely to cause changes in DNA methylation.*

*These information was included in the manuscript (Lines 742-766).*

Fig 1, CDS as black box. But the NTD conserve domains are also indicated as black boxes. This should be changed.

*Reply: We apologize for the confusion. In the revised figure, we added the start codon (ATG) and stop codon (TAA) in the figure to make it clearer.*

Fig S1C: the x-axis labels were missing, but with descriptions in legends such asu embryos (10-38 DAP) or Other tissues. I would suggest to re-make this into multiple panels with better labeling of sample stages and tissue source. This is an important information on how the 4 genes are expressed among different stage or tissue. For example, BS-seq samples were done using 14 DAP embryo and endosperm because of high expression of both genes at this stage (lines 177-180).

*Reply: Thanks for the suggestion, we split the original figure into four figures (Additional file 1: Figure S1C). Hopefully it looks clearer now.*

Which generation of homozygous mutants were used for methylome study?

*Reply: Please refer to major concern 1.*

Fig S2A: I am curious why ZmROS1b expression is reduced in zmros1a mutant embryo? Also what test was done here should be given.

*Reply: We agree that this is an interesting observation. This may reflect potential regulation of some ROS1 orthologs by others but we have not assessed this in detail. The statistical comparisons in Figure S2A utilize the student’s t-test of three replicates of qRT-PCR data. These information has been added in the manuscript (Lines 1165-1166).*

Lines 168-175: I am puzzled if zmros1ab has poorer seed-setting rate, why the allele transmission rate is as expected? I assume the p value was from chi square test, right? Also the genotyping was done in kernels? Or in seedlings? Fig S2C-G, is it possible to assess seed set rates for each mutant? I believe this can be informative. The point is to clarify whether double mutant has gametophytic or early zygotic defects that were missed.

*Reply: We apologize for the confusion. Yes, the P value is from chi square test, and genotyping was done in kernels (using endosperm). The transmission experiment was performed using the self-pollinated ear of heterozygous plants. We self-pollinated the heterozygous plants and genotyped the kernels from the selfed ear. The number of homozygous single/double mutant is observed at the expectation at these self-pollinated ears. The poorer seed set was observed in the self-pollinated ear of the homozygous mutant plant. We think that the double mutant plants are somewhat less vigorous (they also have reduced plant height). This reduced vigor likely is also associated with reduced seed weight even though all genotypes are transmitted at the expected rate.*

Fig 1C: the CHH panels y-scale should be changed to reveal CHH details. It is impossible to see any minor differences in current figure. This applies to Fig S4 as well. CHH methylation is an important readout for RdDM activity and could inform DME-like activity in the maize embryo and endosperm.

*Reply: Thanks for the nice suggestion. We have revised the relevant figures (Figure 1C, S4, and S5) as suggested.*

Fig S4: for embryo CG gene metaplot, why the zmros1b causes reduced in coding sequence CG methylation whereas zmros1a (which expressed at higher level than ZmROS1b in embryo) has almost no effect? In endosperm CG metaplot, why both single mutants cause CG methylation reduction whereas double mutant has elevated methylation?

*Reply: We apologize for the misleading of the figures. Please refer to major concern 2.*

Lines 195-197 and Fig S5: I have a very hard time seeing any meaningful differences between these TEs. The authors should at least point out which ones they were referring to. Perhaps blowup relevant ones in Fig S5 to draw readers’ attention to make their point.

*Reply: We apologize for the confusions. We added red stars to the plots that show methylation changes between WT and mutant. We also added details in the legend to guide the readers. Hopefully it is clear now.*

Lines 210-213: I wonder why this is the case for endosperm methylome if ZmROS1a is not/lowly expressed in the endosperm and zmros1a does not yield too many DMRs. My point is that the statement here seems overly generalized. There could be alternative explanation other than redundancy, at least in the endosperm that worth looking into. For example, Fig S6A endosperm CG DMRs, there are 6000 amros1a-specific DMRs, what’s their methylation levels in wt and in single mutants?

*Reply: This is a nice suggestion and we apologize for not making it clear in the original manuscript. As suggested in the comment, we looked at the methylation levels of the zmros1ab-specific DMRs in WT and in single mutants, DNA methylation levels were increased in both single mutants but didn’t reach our criteria to be called as DMRs. We added the sentence in the manuscript to make it clear (Lines 210-213).*

Lines 219-221: I am having a hard time understanding what the message the authors were trying to convey here. Fig S6C shows there are partial overlaps between CG and CHG DMRs. What does this even mean biologically? This kind of presentation is descriptive and relatively meaningless without providing rational of the comparison.

*Reply: We apologize for the unclarity. We just wanted to show that some regions show increased methylation in several sequence contexts in zmros1ab mutant. This suggests that ROS1 can demethylate more than one sequence context at a particular region. But we don’t have further evidence. To minimize confusion, we deleted the texts and the corresponding figure.*

Lines 236-238: what’s the point of this convoluted and very confusing statement? They are shared DMRs, why do they have to exhibit a reduced methylation relative to embryo??

*Reply: We apologize for the confusion. The shared DMRs show difference in the comparison between WT_endosperm and WT_embryo, as well as in the comparison between WT_endosperm and zmros1ab_endosperm. For these DMRs, we think that the reduced DNA methylation in WT endosperm compared to WT embryo is mediated by ZmROS1ab, as mutation of zmros1ab abolished these DMRs. We revised the texts (Lines 234-245) and hopefully it looks clear now.*

Lines 242-245: I believe a reasonable interpretation of the result is that another DNA glycosylase (most likely ZmROS1d, per Gent et al 2021 BioXriv) functions in maize central cell/endosperm.

*Reply: We agree and added this possibility in the revisions (Lines 249-251).*

Fig S7B: what’s the y-axis unit for log10 transformation?

*Reply: The unit is bp, we added this in the figure.*

Fig S8B: what is the y-axis unit?

*Reply: The unit is “x 1000 normalized read counts”. The normalization was performed in DEseq. We added this in the figure.*

Fig 3C: x-axis label (tissues and stages) should be given

*Reply: Thanks for this suggestion. We have labeled the tissues and stages, and added descriptions in the legend.*

Fig 3D-E: this is an interesting way of assessing endosperm-preferred or -specific expression. However, current description of the analysis provided in the Methods was rough and the figure label is confusing. The x-axis has an increasing label of endosperm-specific expression, ranging from 0.5 to 0.9? What is the y-axis %? I am trying to guess each column represent a list of genes with certain % of endosperm specificity? And among them, some % of them are associated with DMRs? I think this needs to be better presented. The # of genes in each category should be given. Also, the analysis results (all genes with expression score or ratio) should be included as a supplemental file.

*Reply: Thanks for your interest and we apologize for the confusion. The x-axis represent how preferential a gene is expressed in endosperm (see the score in Additional file 6), with low to high preference from the left to the right. These genes (all having a zmros1ab DMR) were divided into 6 categories (shown as 6 columns in the figure), and the y-axis shows the percentage of differentially expressed genes (Figure 3D) or down-regulated genes (Figure 3E) for each category. We revised the labels for the X-axis and added gene number for each column; we revised the legend to make it clear.*

Shouldn’t a parallel set analysis be done for embryo genes as well as a control?

*Reply: This is a nice suggestion. We performed similar analysis and found 29 CG DMRs and 43 CHG DMRs that are zmros1ab-sensitive DMRs in embryo (hypomethylated in embryo compared to endosperm and hypermethylated in zmros1ab embryo compared to WT embryo). These DMRs were associated with 13 genes, none of which is embryo-specific expressed. This has been added in the manuscript (Lines 319-325).*

Fig 3F: plots should be enlarged.

*Reply: Thanks for the suggestion. The figure has been enlarged.*

Fig 3G: this looks very nice. I would very much like to see single mutant methylation tracks added. And if possible, should a few more examples in a supplemental figure.

*Reply: Thanks for the nice word. We have added the tracks for the single mutant and included more examples in the supplemental figure (Additional file 1: Figure S8E).*

Line 315: should specify in maize endosperm. If in Arabidopsis, a whole different set of references should be included here.

*Reply: Thanks for the suggestion. We have added the word “maize” (Line 337).*

Fig 5A and B: the main text is a bit confusing and seems different from Fig legend. Endo-MEGs are the 17 MEGs differentially expressed (line 341)? Perhaps giving gene numbers (N=xx) will help with clarification. Perhaps Fig 5A&B should be moved to supplementals?

*Reply: We apologize for the confusion. Yes, these are the 17 Endo-MEGs. We have revised the legend to make this clear. We also added gene number in the figure. And these two figures have been moved to supplemental (Additional file 1: Figure S9C-S9D).*

Fig S9C&D: it will be nice to indicate where is the SNPs and Chop-PCR primers are located (promoter, coding, etc).

*Reply: Thanks for the suggestion. We have added a gene model with the SNPs and primers labeled.*

Line 451-452: maize should be included in the sentence here. If also include Arabidopsis, a whole new set of references should be added.

*Reply: Thanks for the suggestion. The word “maize” has been added (Line 466).*

Line 461: cite the Fig for easy reference.

*Reply: Thanks for the suggestion. Figure 2B has been cited (Line 477).*

Line 468: change specifically expressed to preferentially expressed for better accuracy.

*Reply: Thanks for the suggestion. This has been revised as suggested (Line 487).*

Line 470: pretty sure Park 2017 isn’t the right reference for this statement. I think Ibarra et al 2012, Slotkin et al 2009, and Kinoshita et al 2004 should be included as well.

*Reply: Thanks for pointing this out. The Park 2017 paper was replaced by the suggested papers (Lines 489-490).*

Line 476: is Opague2 a known imprinted gene? If so, cite reference. I think the choice of Opague2 for DAP-seq can be better rationalized in the DAP-seq result section.

*Reply: Thanks for this consideration. Opaque2 is not an imprinted gene.*

Lines 505-507: ZmROS1b is highly expressed in endosperm. I think the discussion here should not ignore this fact.

*Reply: We agree, and added this in the revised manuscript (Lines 526-527).*

Line 509: Part et al 2017 is not the right reference for here. Did you mean Park 2016? I think Hsieh et al 2011 PNAS is a more appropriate reference for this.

*Reply: Thanks for pointing this out, we deleted the Park paper and added the Hsieh paper (Lines 529-530).*

Lines 505-506: PMID: 34185870, 26400170, 30589221 can be added to strengthen your reasoning.

*Reply: Thanks for the suggestion. These references have been added (Lines 573-574).*

Lines 560-: Suggest changing to “The methylation changes in zmros1ab mutant can affect transcription factor binding…..”

*Reply: Thanks for the suggestion; we have revised the texts as suggested (Line 584).*

**Third round of review**

**Reviewer 3**

The authors have addressed most of the concerns I have raised. However, one major issue regarding the background mutations still exists. I appreciate their effort to try to assess the spectrum of background mutations present in the mutant lines by analyzing RNA-seq reads. The potential effect of any uncharacterized or unidentified mutations, particularly in the regulatory elements, cannot be completely ruled out. Consequently, the observed difference in various genomic profiles between wt and mutants cannot be unequivocally ascribed as the function of these demethylases. There are numerous prior examples of phenotypes caused by unnoticed background mutations there were not aware in primary publications (e.g., Plant Physiol 2020 Jan;182(1):460-462. Nat Plants. 2015;1:15183) highlighting the importance of using a clean mutant background to the best knowledge of the authors for important study such as this work. I strongly recommend the authors to clean up the mutant lines and repeat the experiments and analyses.

**Reviewer 1**

Cleaning up the mutation in maize is going to take years. I am inclined to let the authors publish the paper while adding the caveat about the cryptic mutation. Apart from that, the other thing I feel the authors can do is to use their WGBS data to see if they can find mutations in ROS1 paralogs.

Overall, I feel comfortable to publish this paper.

**Authors Response**

**Point-by-point responses to the reviewers’ comments:**

Reviewer #3:

The authors have addressed most of the concerns I have raised. However, one major issue regarding the background mutations still exists. I appreciate their effort to try to assess the spectrum of background mutations present in the mutant lines by analyzing RNA-seq reads. The potential effect of any uncharacterized or unidentified mutations, particularly in the regulatory elements, cannot be completely ruled out. Consequently, the observed difference in various genomic profiles between wt and mutants cannot be unequivocally ascribed as the function of these demethylases. There are numerous prior examples of phenotypes caused by unnoticed background mutations there were not aware in primary publications (e.g., Plant Physiol 2020 Jan;182(1):460-462. Nat Plants. 2015;1:15183) highlighting the importance of using a clean mutant background to the best knowledge of the authors for important study such as this work. I strongly recommend the authors to clean up the mutant lines and repeat the experiments and analyses.

Reviewer #1:

Cleaning up the mutation in maize is going to take years. I am inclined to let the authors publish the paper while adding the caveat about the cryptic mutation. Apart from that, the other thing I feel the authors can do is to use their WGBS data to see if they can find mutations in ROS1 paralogs.

Overall, I feel comfortable to publish this paper.

*Reply: Thanks for the suggestion. We understand the concern and agree that we cannot completely rule out the potential effect of any uncharacterized or unidentified mutations. As purifying the mutant lines is impractical at this stage, we tried to identify the mutations in ZmROS1 paralogs using our WGBS data. Besides, we discussed the potential issue of cryptic mutations in the revised manuscript.*
